# Supplementary material for: Development of a core outcome set for evaluative research into paediatric cerebral visual impairment (CVI), in the UK and Eire
Source: BMJ Open. 2021 Sep 29;11(9):e051014. doi: 10.1136/bmjopen-2021-051014 (PMC8483040; doi:10.1136/bmjopen-2021-051014)
Supplement: Supplementary data [file bmjopen-2021-051014supp001.pdf]

University of BRISTOL

Research Protocol

The CVI Project: A Core Outcome Set for children with Cerebral Visual impairments (COS-CVI)

THE CVI PROJECT

NHS National Institute for Health Research

Cathy Williams
& Anna Pease

Contents

Project Team 3
A Core Outcome Set for Children with Cerebral Vision Impairments (COS-CVI) 4
Background 5
Methods 6
Scope 6
Public Involvement 6
Phase 1. Identifying Candidate Outcomes 6
Proportionate Systematic Review 6
Qualitative work 7
Phase 2. Rating the importance of Candidate Outcomes using a Delphi Survey 9
Panels 9
Software, nature and ordering of questions 9
Number of rounds and nature of feedback given 9
Consensus workshop 10
Discussion and implementation 11
Gantt Chart for Core Outcome Set Development 12
Figure 1: Core Outcome Set Development Flowchart 13
References 14

Pease A, *et al. BMJ Open* 2022; 11:e051014. doi: 10.1136/bmjopen-2021-051014

Project Team

Ms Cathy Williams
Post: Reader in Paediatric Ophthalmology
Email: Cathy.Williams@bristol.ac.uk
Dept: Centre for Child and Adolescent Health, School of Social and Community Medicine
Address: University of Bristol, Oakfield House, Oakfield Grove, Bristol, BS8 2BN
Tel: 0117 3313388

Dr Anna Pease
Post: Senior Research Associate
Email: A.pease@bristol.ac.uk
Dept: Centre for Child and Adolescent Health, School of Social and Community Medicine
Official Address: University of Bristol, Oakfield House, Oakfield Grove, Bristol, BS8 2BN
Tel: 0117 331 3310

Dr Trudy Goodenough
Post: Senior Research Associate
Email: Trudy.Goodenough@bristol.ac.uk
Dept: Centre for Child and Adolescent Health, School of Social and Community Medicine
Official Address: University of Bristol, Oakfield House, Oakfield Grove, Bristol, BS8 2BN
Tel: 0117 331 3310

Ms Rose Watanabe
Post: Executive Assistant
Email: rose.watanabe@bristol.ac.uk
Dept: Centre for Child and Adolescent Health, School of Social and Community Medicine
Official Address: University of Bristol, Oakfield House, Oakfield Grove, Bristol, BS8 2BN
Tel: 0117 331 4005

Dr Christopher Morris
Post: Senior Research Fellow
Email: Christopher.Morris@exeter.ac.uk
Dept: Child Health Group Peninsula Cerebra Research Unit for Childhood Disability Research (PenCRU)
Official Addess: University of Exeter Medical School, South Cloisters, St Luke's Campus, Exeter, EX1 2LU
Tel: 01392 262980

Ms Cath Borwick
Post: Research Engagement Librarian - Medical Faculties
Email: cath.borwick@bristol.ac.uk
Dept: Medical Library
Official Address: Medical Sciences Building, University of Bristol, Bristol BS8 1TD
Tel: (0117) 331 1504 or 394 1079

Funding Source: National Institute of Health Research (NIHR) Senior Research Fellowship Award

A Core Outcome Set for Children with Cerebral Vision Impairments (COS-CVI)

Abstract

In the current climate of financial challenges and limited resources, the importance of best use of available funds is paramount. Core outcome sets are condition-specific sets of outcomes that are developed by and represent the concerns of a range of stakeholders including patients, carers, professionals and policy makers. The aim of this project is to develop a COS for use in evaluative research on interventions that aim to improve outcomes for children with brain-related vision impairments, commonly known as cerebral visual impairments or CVI.

Methods

This project is nested within a 5-year programme of work investigating the prevalence, impact and effectiveness of support for primary school age children with CVI. We will use methods recommended by the COMET Initiative (Core Outcome Measures in Effectiveness Trials) including a proportionate review of what's been measured before; qualitative work to bring in the voices of children and families; a Delphi survey involving parents, children, professionals in medicine and teaching and therapists and a consensus meeting to determine the most important outcomes. We will then review the available tools and decide how best to measure the selected outcomes.

Discussion and Implementation

Our methods are proportionate to the resources available in the 5-year programme and we will reflect and discuss any consequent limitations of this on our findings. We will then use the COS and the tools that best measure the selected outcomes in a feasibility cluster-randomised controlled trial (fcRCT). Outcomes from the fcRCT will include the acceptability and practicality of the COS and these may be useful for further development of the COS.

Pease A, *et al. BMJ Open* 2022; 11:e051014. doi: 10.1136/bmjopen-2021-051014

Background

Brain-related vision impairments, commonly known as cerebral visual impairments or CVI, are frequently reported in studies of patients who have suffered a form of injury to their central nervous syndrome or who are born with an abnormality in the brain structure or function.

CVI is an umbrella term used by many to include abnormalities in visual acuity, or contrast sensitivity or colour; ocular motility; visual field and the conscious and unconscious filtering or processing of visual input (1). However, in the current World Health Organisation (WHO) classification of diseases ICD10, only codes for acuity and field loss are used in the description of "visual impairment (VI)" and consequently in many countries, including the UK, certification of VI involves only those visual functions (2). There is not as yet a consensus within the UK or internationally as to how other impairments of visual functioning, such as eye movements or vision processing, should be characterised. The new ICD classification ICD11 is likely to contain addition codes for complex vision impairment affecting functions such as reading or navigation and this may aid future research and resource allocation (3). In the meantime, there are very few studies to guide parents or vision professionals or therapists on how much of a person's difficulties might be attributable to CVI, nor on how best to help them.

Individuals most at risk of CVI are those with childhood-onset neurodevelopmental disorders (1), and adults who develop neurological disorders particularly including stroke and dementia (4). Some of the problems relating to having CVI are likely to be present at all ages. Children with CVI have additional specific needs and problems relating to their development from infancy to adulthood and their need for education whilst adults who acquire their neurological problems later in life may have different problems relating to employment and adjustment to their new impairment.

Currently there are 17 projects registered in the "Eyes and Vision" section of the COMET database (5). All but two of them focus on adults rather than children. One of the adult projects is however specifically on CVI after stroke and will be contacted as there may be overlap between child and adult outcomes after CVI.

As part of establishing to what extent children with CVI might be helped by interventions in a future RCT, we will therefore develop a specific COS for children with CVI. We will take an inclusive approach and include CVI however diagnosed, to reflect (a) the current lack of consensus about what brain related impairments should be regarded as visual and (b) variation in availability of detailed vision testing to elicit all the potential manifestations of CVI.

Pease A, et al. BMJ Open 2022; 11:e051014. doi: 10.1136/bmjopen-2021-051014

Methods

We will register the study with the COMET Initiative. We will seek ethical committee permission through the University Faculty Research Ethics Committee (FREC).

Scope

We will include children of school-age up to 18 as they will be able to contribute their perspectives relating to their own recent past as well as their present, although the future fcRCT will be focussing on primary school children. We will consider outcomes of interventions to help children with CVI in any setting, including evaluative trials and routine practice in hospitals and schools. Our primary aim however is to arrive at an agreed COS for use in the planned fcRCT. We will use the PICO format to specify the scope of the study. The scope is participants in the UK and Ireland, though broader generalizability internationally could be explored subsequently.

Public Involvement

We already have had input from a local group of families whose children have CVI and from the PenCRU "Family Faculty" coordinated by University of Exeter. These are a group of parents whose children have a range of neurodevelopmental conditions. We will ask their advice on practical details of interacting with children with neurodevelopmental conditions in the planned qualitative interviews. They will also be consulted on interpreting the results and disseminating the findings.

Phase 1. Identifying Candidate Outcomes

Proportionate Systematic Review

We will review the literature to see which outcomes have been measured in interventions to help children with CVI.

Search strategy
We will include directly assessed, patient-reported and proxy-reported outcomes for children with CVI according to any definition made by a relevant professional (eg a vision specialist, a neuropsychologist or a doctor.

Types of Studies
Any interventional study that aims to improve outcomes for children with CVI as defined above

Types of Participants
Children up to 18 with CVI as defined above

Inclusion criteria

We will include studies that report on children with what the study reports as CVI, however defined. We will include experimental studies and observation studies that describe associations with CVI defined by a relevant professional

Exclusion criteria

We will exclude studies where it is not possible to separate adult from child data, where there is no description of the visual behaviour used to define the child as having CVI and studies not in English.

Data extraction

We will record each type of outcome recorded in included papers and the instrument used to measure or ascertain the outcome. We will record study type and participant characteristics as well as numbers of respondents and non-respondents, or non-applicable responses, for each outcome. Data will be extracted by two reviewers and compared and consensus reached by discussion with a third reviewer.

Synthesis

We will list all outcomes from the papers in a database as well as the instruments used. We will code and organize them in two ways. Firstly, we will use the forthcoming taxonomy proposed by the COMET Initiative (6). Secondly, we will map them to the domains in the WHO International Classification of Functioning (ICF) insofar that is possible. These two models will be used to sort the outcomes as per Wilson and Cleary (7) into biological or functional measures; activity or participation measures, mental health states and quality of life.

We will submit draft coding and lists to our advisory panels for discussion and refinement.

Qualitative work

Aim

The aim of the qualitative work will be to “fill in the gaps” revealed by the systematic review and capture the language and perspectives of children and their families.

Participants

Participants will be children and young people aged 6 - 18 years with a diagnosis of CVI, using any definition, and/or parents or carers of children with this diagnosis.

Sampling method
To include a range of ages and physical capabilities, maximum variation purposive sample will be used to recruit families to a matrix including younger (6-11 years old) and older (12-18 years old) children, and those with and without a diagnosis of cerebral palsy.

Recruitment
We will recruit children and young people from three sources initially and will use a snowballing approach to recruit more if needed. Firstly, we will ask the local Specialist teachers for Vision Impairment to post a notice with information and contact details on their website. They will also notify individuals they think might be interested. We will visit a local school for children with visual impairment (The WESC Foundation, Specialist Centre for Visual Impairment, Exeter) and distribute notices and information. Thirdly we will give the same information to a national parent support group for families of children with CVI (www.cvisociety.org.uk).

Data collection – interviews
Interviews will be carried out in person, at school or at home as preferred or as convenient. Children will be interviewed with a trusted adult present (this can be a parent or member of school staff), after their parent or carer has been interviewed. All interviews will be audio-recorded with consent. A topic guide based on the systematic review and comments from the advisory groups and based on approaches already reported and successful with similar-aged children will be used (for example an activity discussing "a day in the life of a child with CVI"). Interviews will be expected to take 30-45 minutes for adults and 10-30 minutes for children. When interviewing children a range of creative activities will help us to elicit views of young people about what matters most to them. We will use drawings, stories and a tablet to support these approaches to follow the topic guide.

Interviews will continue with families until the point of diminishing returns, when little or no new perspectives are forthcoming and no new outcome domains are being identified. We expect to conduct interviews with approximately 30 families in total.

Analysis
A framework analysis consisting of five steps will be used. First familiarisation with the data will allow for submersion in the topic area. Secondly an initial framework will be applied by coding key issues in the transcripts. Third, the codes will be indexed into categories, fourth, thematic charts will be produced and compared by independent coders (members of the research team) and finally the charts will be interpreted into themes using key ideas and quotes from the original data.

Mapping to a framework
We will map the data obtained from the qualitative interviews into categories according to the two coding schemes (the COMET taxonomy and the ICF) described above.

Phase 2. Rating the importance of Candidate Outcomes using a Delphi Survey

We will carry out a Delphi survey to rate the importance of the outcomes assembled from the systematic review and the interview data. A Delphi survey is an iterative process of defining and refining importance of outcomes, using several rounds of surveys involving parents, children, professionals in medicine and teaching and therapists. The process ends with a consensus meeting to determine the most important outcomes.

Panels

We will invite individuals to participate in either of two panels:
1. Professionals (ophthalmologists, optometrists, orthoptists, paediatricians, therapists, teachers)
2. Parents and YP and children.

We will ask local and national organisations to advertise the survey to professionals. We will ask local schools, the Specialist teachers and patient support groups to advertise the survey to families including children themselves if they want to participate, potentially with the support of a parent or carer, or independently. We will also invite those families who have taken part in the qualitative work to take part in the Delphi if they wish, but this is not expected or mandatory. We will invite individuals willing to participate to register through an online system (COMET's Delphi Manager) or to contact the study team directly. We will record the professional role, years in role, institution and area of the country for professionals taking part. We will record age, ethnicity and diagnosis/es of affected children for the families taking part.

Software, nature and ordering of questions

We will use DelphiManager (a web based data management system developed by COMET) to facilitate the construction and delivery of our surveys. The software allows data to be extracted and consolidated at every round, as well as providing analysis of responses.

Number of rounds and nature of feedback given

Pease A, *et al. BMJ Open* 2022; 11:e051014. doi: 10.1136/bmjopen-2021-051014

Round 1: In the initial stage of the survey we will present the findings of the systematic review and we will ask respondents to rate the importance of each using a 9 point scale in which 1-3 relates to "less important" and 7-9 relates to very important. We will also ask respondents to add any additional outcomes that they feel should be included. Those items that score a median of 7 or above and/or 70% from either panel rating the outcome as 7-9 will be carried into round 2.

Round 2: Following the initial stage of rating the systematic review findings and generating further outcomes, we will add in the list of outcomes provided through the qualitative work (taking out any overlapping items from each stream) and present the complete list in Round 2. We will ask respondents to rate each item as before. For those items that have been rated previously, respondents will be shown the median scores and range for three groups; (1) the scores from professionals, (2) the scores from families and (3) the overall median score from both panels. Those items that score a median of 7 or above and/or 70% from either panel rating the outcome as 7-9 will be carried into round 3.

Round 3: In round 3, respondents will be shown their score from Round 2 as well as the median score and range from each panel who responded for each item. We will include items where there is agreement between panels that items are important and also items where there is polarisation (e.g. professionals scored high but families scored low) between panels and indicate this in the survey. Space will be provided for respondents to add their own comments on how they have rated items and to add any further considerations.

We will send each final survey for each round to the Faculty of Health Sciences Research Ethics Committee for information.

Consensus workshop

We will hold a consensus workshop with attendees from both panels in Bristol, to ratify agreement of the more important outcomes. We will highlight that the core outcomes are a minimum set for measuring change and that interventions may add in other outcomes as they see fit. We will present a list of outcomes identified in round 3 with a median score of 7 or above with 70% from either panel rating the outcome as 7-9. Research team members will facilitate the workshop. All UK participants will be invited to attend. Attendees will be shown the list of outcomes to be ratified prior to the workshop. The workshop will comprise three parts.

Pease A, *et al. BMJ Open* 2022; 11:e051014. doi: 10.1136/bmjopen-2021-051014

Part 1 will include introductions for participants and introduce the format for the workshop. This will include the aims of the day and the threshold for inclusion of items into the final core set.
Part 2 will see participants discuss and rate the importance of items in mixed stakeholder groups, with a facilitator for each group.
Part 3 will bring the participants back together to arrange the final set of outcomes into importance and will be asked to endorse the proposed final set.

Discussion and implementation

Once we have identified what to include in the fcRCT from the above process, we will review the most feasible way to collect data on these outcomes. This COS project is necessarily proportionate to the resources in the NIHR Fellowship award that funds it and additional work such as the development of new assessment tools, is outside the scope and resources available. We will use the COS obtained from the consensus meeting to inform selection of outcome measures in the fcRCT. We will collect data on the feasibility and acceptability of the data collection for these outcome measures and use this to guide any further developments of a COS for children with CVI.

Timescale

The Gantt chart on page 12 indicates the sequence of events and the originally planned timescale. The qualitative work will extend into July due to staff changes and a

Pease A, *et al. BMJ Open* 2022; 11:e051014. doi: 10.1136/bmjopen-2021-051014

Gantt Chart for Core Outcome Set Development

COS-CVI

Duration

ACTIVITY	2018 May	June	July	August	September	October	ovembe	ecembe	Januar	ebruar	March
**Proportionate Systematic Review**											
Develop search strategy	■										
Screening Titles and Abstracts		■									
Screening Full Texts		■									
Data Extraction			■								
Data Synthesis			■								
**Qualitative Work**											
Recruitment	■	■									
Data collection	■	■	■	■							
Transcription	■	■	■	■							
Coding and Analysis			■	■	■						
Framework Mapping			■	■	■						
**Delphi Exercise**											
Participants invited		■	■	■							
Round 1 rating			■	■							
Round 2 rating				■	■						
Round 3 rating					■	■					
Consensus Workshop						■	■				
Framework Mapping							■				
**Dissemination of findings**											
PPI meetings							■	■	■	■	■
Writing up of report							■	■	■	■	■
Paper submission							■	■	■	■	■
Conference Presentations							■	■	■	■	■

Pease A, *et al. BMJ Open* 2022; 11:e051014. doi: 10.1136/bmjopen-2021-051014

Figure 1: Core Outcome Set Development Flowchart

Phase 1: Proportionate Systematic Review

- Identify outcomes in RCTs involving children with CVI

Phase 2: Qualitative Interviews

- Capture perspectives and language of children with CVI and their families

Phase 3: Delphi Exercise

- Rate the importance of candidate outcomes
- Two panels - professionals and families

Round 1

1. Present outcomes from systematic review and rate on likert scale from 1 to 9
2. Provide feedback on ratings (optional)
3. Suggest new outcomes

Round 2

Include Outcomes which score a median of 7 or above and/or 70% from either panel rating the

1. Present outcomes from round 1 and qualitative interviews
2. Participants see own score, median for each panel and overall median score
3. Re-rate items from 1-9
4. Provide feedback on ratings (optional)

Round 3

Include Outcomes which score a median of 7 or above and/or 70% from either panel rating the

1. Present outcomes from round 2
2. Participants see own score, median for each panel and overall median score
3. Re-rate items from 1-9
4. Provide feedback on ratings (optional)

Consensus Workshop

1. Introductions and aims for the workshop
2. Break out groups to discuss and rate items
3. Consensus reached on core outcome set

Dissemination and Implementation

1. Report writing
2. Data collection methods for core outcomes reviewed
3. Select outcome measures for future fcRCT

Fig 1. Core Outcome Development Flowchart. Adapted from Tong et al. (2016)

Pease A, *et al. BMJ Open* 2022; 11:e051014. doi: 10.1136/bmjopen-2021-051014

References

1. Philip, S. S. and Dutton, G. N. (2014), Identifying and characterising cerebral visual impairment in children: a review. Clin Exp Optom, 97: 196–208. doi:10.1111/cxo.12155

2. https://www.gov.uk/government/publications/guidance-published-on-registering-a-vision-impairment-as-a-disability

3. https://icd.who.int/dev11/proposals/

4. Visual Perceptual Consequences of Stroke Fiona Rowe & VIS Group UK. Strabismus Vol. 17, 1,2009

5. http://www.comet-initiative.org

6. Trials. 2017 Jun 20;18(Suppl 3):280. doi: 10.1186/s13063-017-1978-4. The COMET Handbook: version 1.0.

7. Wilson IB, Cleary PD: Linking clinical variables with health-related quality of life: a conceptual model of patient outcomes. JAMA 1995, 273(1):59-65.
