# Supplementary material for: Development of a core outcome set for evaluative research into paediatric cerebral visual impairment (CVI), in the UK and Eire
Source: BMJ Open. 2021 Sep 29;11(9):e051014. doi: 10.1136/bmjopen-2021-051014 (PMC8483040; doi:10.1136/bmjopen-2021-051014)
Supplement: Author's manuscript [file bmjopen-2021-051014.draft_revisions.pdf]

BMJ Open

BMJ Open is committed to open peer review. As part of this commitment we make the peer review history of every article we publish publicly available.

When an article is published we post the peer reviewers' comments and the authors' responses online. We also post the versions of the paper that were used during peer review. These are the versions that the peer review comments apply to.

The versions of the paper that follow are the versions that were submitted during the peer review process. They are not the versions of record or the final published versions. They should not be cited or distributed as the published version of this manuscript.

BMJ Open is an open access journal and the full, final, typeset and author-corrected version of record of the manuscript is available on our site with no access controls, subscription charges or pay-per-view fees (http://bmjopen.bmj.com).

If you have any questions on BMJ Open's open peer review process please email info.bmjopen@bmj.com

BMJ Open

Development of a core outcome set for Paediatric Cerebral Visual Impairment (CVI)

Journal:	*BMJ Open*
Manuscript ID	bmjopen-2021-051014
Article Type:	Original research
Date Submitted by the Author:	06-Mar-2021

Complete List of Authors:	Pease, Anna; University of Bristol,<br>Goodenough, Trudy; University of Bristol, Bristol Medical School<br>Borwick, Cath; University of Bristol<br>Watanabe, Rose; University of Bristol<br>Morris, Christopher; University of Exeter Medical School<br>Williams, Cathy; University of Bristol

Keywords:	Paediatric ophthalmology < OPHTHALMOLOGY, EPIDEMIOLOGY, QUALITATIVE RESEARCH

SCHOLARONE™
Manuscripts

I, the Submitting Author has the right to grant and does grant on behalf of all authors of the Work (as defined in the below author licence), an exclusive licence and/or a non-exclusive licence for contributions from authors who are: i) UK Crown employees; ii) where BMJ has agreed a CC-BY licence shall apply, and/or iii) in accordance with the terms applicable for US Federal Government officers or employees acting as part of their official duties; on a worldwide, perpetual, irrevocable, royalty-free basis to BMJ Publishing Group Ltd ("BMJ") its licensees and where the relevant Journal is co-owned by BMJ to the co-owners of the Journal, to publish the Work in this journal and any other BMJ products and to exploit all rights, as set out in our licence.

The Submitting Author accepts and understands that any supply made under these terms is made by BMJ to the Submitting Author unless you are acting as an employee on behalf of your employer or a postgraduate student of an affiliated institution which is paying any applicable article publishing charge ("APC") for Open Access articles. Where the Submitting Author wishes to make the Work available on an Open Access basis (and intends to pay the relevant APC), the terms of reuse of such Open Access shall be governed by a Creative Commons licence – details of these licences and which Creative Commons licence will apply to this Work are set out in our licence referred to above.

Other than as permitted in any relevant BMJ Author's Self Archiving Policies, I confirm this Work has not been accepted for publication elsewhere, is not being considered for publication elsewhere and does not duplicate material already published. I confirm all authors consent to publication of this Work and authorise the granting of this licence.

For peer review only

Development of a core outcome set for Paediatric Cerebral Visual Impairment (CVI)

Pease, A[1], Goodenough, T[1], Borwick, C[1], Watanabe, R[1], Morris, C[2], and Williams, C[1],

1.Bristol Medical School, 1-5 Whiteladies Road, University of Bristol, Bristol BS8 1NU, UK

2.PenCRU (Peninsula Childhood Disability Research Unit), University of Exeter Medical School, University of University, UK

Corresponding Author

C Williams
1-5 Whiteladies Road
Clifton
Bristol
BS8 1NU
Cathy.Williams@bristol.ac.uk

Main text words = 3910

For peer review only

ABSTRACT

Objectives

Cerebral visual impairment (CVI) comprises a heterogenous group of brain-related vision problems. A core outcome set (COS) represents the most important condition-specific outcomes that reflect the priorities of patients, carers, professionals and researchers. Sets are used to facilitate synthesis across studies. We aimed to produce a COS for studies evaluating interventions for children with CVI, to increase the relevance of research for families and professionals and help develop the evidence-base.

Design

We used methods recommended by the COMET (Core Outcome Measures in Effectiveness Trials) Initiative. These included a proportionate literature review of outcomes used in previous studies; qualitative interviews with children and families; a two-round Delphi survey involving parents, children and professionals and a consensus meeting to ratify the most important outcomes.

Setting

Telephone interviews and online Delphi survey

Participants

Eighteen parents and 6 young people were interviewed. Delphi participants (n=80 for both rounds) included professionals working with children who have CVI (teachers, orthoptists, ophthalmologists, optometrists, qualified teachers for visually impaired (QTVI), family members (parents and siblings) and affected children.

Results

The literature review included 13 studies yielding 37 outcomes. Qualitative interviews provided 22 outcomes. After combining and refining similar items, the first round contained 23 outcomes and the second 46. the consensus meeting, five attendees recommended 28 outcomes for inclusion in the CVI COS, of which 15 were ratified as most important, including 4 related to vision; 1 to family wellbeing; 1 to adults around the child being informed about CVI and the rest to the child's abilities to engage with people and surroundings.

Conclusions

Good engagement from participants led to the development of a COS. Future research will be useful to identify the best ways to measure COS items and potentially to update this COS as more interventions for CVI are developed.

295 words

Keywords: CVI, Children, Core outcome set, Paediatric

For peer review only

ARTICLE SUMMARY

Strengths and Limitations of the study

Methods advocated by the COMET Initiative were used to propose a core outcome set for evaluative trials and routine practice in hospitals and schools on childhood cerebral visual impairment (CVI)

Parents, children and professionals took part in the process as interviewees, Delphi participants and/or consensus meeting attendees

Several outcomes included in the final COS were originally suggested by participants (both family members and professionals)

The COS may not reflect the views of researchers or journal editors who were not involved.

Several outcomes were broad concepts and further research is needed to determine how to measure the COS.

For peer review only

INTRODUCTION

Cerebral Visual Impairment (CVI) refers to a range of brain-related impairments of vision. Although there is not yet an agreed definition for CVI, a recent review has summarised the literature and suggested that CVI be regarded as a verifiable impairment of vision that is not attributable to ocular or anterior visual pathway (optic nerve) disorders.[1] However different groups and centres take different views on which visual disorders can be described as CVI and in particular there is variation in the levels of visual acuity loss that different authors describe as consistent with CVI [1]. There are not at present and nationally or internationally agreed guidelines for the management of CVI in children.

A core outcome set (COS) represents the most important condition-specific outcomes that reflect the priorities of patients, carers, professionals and researchers. Further work is then required to establish "how" to measure the items in the COS. Use of a COS ensures research findings are relevant to health service users, ie patients and carers, as well as to healthcare professionals. A COS facilitates synthesis across studies and aggregation of data in meta-analyses or evidence syntheses, to develop a robust evidence base [2].

Guidelines exist to aid researchers in developing core outcome sets (CO_STAD[3])and reporting processes (CO-STAR[4]), which we have followed insofar as our resources allowed. The COMET (Core Outcome Measures in Effectiveness Trials) database describes established and ongoing COS and did not previously include a COS for CVI. The COMET recommended methods [5] include a literature review to discover which outcomes have already been measured in relevant studies, qualitative research to elicit the views of patients, families and carers; a Delphi survey (an online, iterative survey with anonymous participants [6]) in which stakeholders from patient and professional groups rated outcomes by importance and finally a consensus meeting involving representatives of all stakeholder groups to discuss and ratify the final items in the COS. These methods have recently been used to produce COS for assessment of brain-related impairments of vision in adults who have suffered a stroke[7]. We aimed to produce a COS for use in evaluative studies involving children with CVI.

METHODS

Scope

This COS relates to children aged up to 18 years in the UK and Ireland, relating to interventions to help children with CVI in any setting, including evaluative trials and routine practice in hospitals and schools. We took an inclusive approach and included CVI as a broad concept, however diagnosed, to reflect (a) the current lack of consensus about which brain-related impairments should be included, and (b) variation in availability of detailed vision testing to elicit all the potential manifestations of CVI.

Ethical Approval

The study was registered with the COMET Initiative (http://www.comet-initiative.org/studies/details/1032). The study was approved by the University of Bristol Faculty of Health Sciences Research Ethics Committee (FREC ref: 58441). Written consent was documented for the interviews, from both parents and young people. Consent to the Delphi survey was implied by the completion and return of the questionnaires.

Patient and Public Involvement

For peer review only - http://bmjopen.bmj.com/site/about/guidelines.xhtml

We used a range of methods to involve patients and the public and these are summarised in Box 1, using the GRIPP2 recommended headings [8]. We convened meetings with two group of families: one in the Bristol area whose children have CVI and the other with the PenCRU Family Faculty at University of Exeter Medical School. The PenCRU Family Faculty comprises parents whose children have various neurodevelopmental conditions and who regularly contribute to and advise on research studies. We sought their advice on practical details of interacting with children with neurodevelopmental conditions in the planned qualitative interviews. We presented our plans at the yearly meeting of a family support group for children with CVI (the CVI Society, www.cvisociety.org.uk) and families were invited to participate in the qualitative interviews or Delphi survey and/or invite other people to participate. Study details were posted on the CVI Society website and in their closed Facebook group.

We met with our Professional Advisory Group which includes education and health professionals with an interest in CVI. They advised on the best ways to contact professionals e.g. by using dedicated email lists. Parents and 3 young people with CVI then advised on the wording of the outcomes included in the Delphi survey.

Stage 1: Literature Review

A proportionate review of the literature was carried out to identify outcomes that have been measured in evaluations of interventions to help children with diagnosed CVI or with brain-related vision impairments if the term CVI was not used. A search strategy was updated from a previous scoping review on interventions for CVI, [9] and the resulting set of papers was searched specifically for papers reporting controlled studies, before-and-after studies or service evaluations, involving children with CVI using any definition or brain-related vision problems. Searches were completed in Medline, Embase, PsychInfo and the Cochrane database of systematic reviews (search dates 1946 – May 2018). Papers that reported on eligible studies involving children up to 18 years were included, including papers reporting methods before results were obtained, since these contained outcomes relevant to the purpose of our review. Papers where it was not possible to separate adult from child data, where there was no description of the visual behaviour used to define the child as having CVI or brain-related vision problems and papers not in English (as no translation funds were available), were excluded.

A data extraction form was used to record all outcomes used in each included paper, whether primary or secondary, the instruments used, the study type and setting, participant characteristics, and data completion for each outcome. Also included was which domain the outcome mapped to, using the COMET taxonomy[10]. Data were independently extracted by two reviewers (CW and TG) and consensus was reached by discussion with a third reviewer.

Stage 2: Qualitative interviews with families where a child has CVI

Participants were children and young people (CYP) aged 6 - 18 years with a diagnosis (by a professional) of CVI, using any definition, and/or parents or carers of children with this diagnosis. To include a range of ages and physical capabilities, maximum variation purposive sampling was used to recruit families to a matrix including younger (6-11 years old) and older (12-18 years old) children, and those with and without a diagnosis of cerebral palsy. Families were recruited from three sources: from local Specialist teachers for Vision Impairment; from The Specialist Centre for Visual Impairment, Exeter (WESC Foundation), and from the CVI Society, a national parent support group for families of children with CVI (www.cvisociety.org.uk) as described above. Recruitment was also facilitated by snowballing information to other interested groups via from the initial contacts, including links to 'Moorvision' (https://www.moorvision.org/) via a local specialist school (the WESC Foundation specialist school for visual impairment).

Interviews were carried out in person either face-to-face or by telephone or video link. Parents were present for interviews with young people. All interviews were audio-recorded with consent. Topic guides

For peer review only - http://bmjopen.bmj.com/site/about/guidelines.xhtml

covered what day-to-day family life living with CVI is like, including school, home, family and health and wellbeing; practicalities of living with CVI; support received and what would have helped in the past and what would help in the future. For interviews with young people, a range of creative activities were used to help elicit their views about what matters most to them, including drawing, colouring and tablet-based art activities. Interviews continued until the point of diminishing returns, when little or no new perspectives were forthcoming and no new outcome domains were being identified.

The interviews were transcribed verbatim and systematic coding across the transcripts was used to identify outcomes for inclusion in the Delphi Survey. The full thematic analysis of these interviews exploring the impact of CVI on the daily lives of children and their families will be reported separately.

Stage 3: Delphi Process

A Delphi survey[6] was used for participants to rank the importance of the outcomes assembled from the systematic review and the interview data. There were two rounds to the survey and a subsequent face-to-face consensus meeting to ratify agreement on the most important outcomes.

Two groups of participants were invited to take part in the Delphi survey: professionals and families. Professionals included ophthalmologists, optometrists, orthoptists, paediatricians and specialist teachers (Qualified Teachers for Visual Impairment, QTVIs). Families included parents of children with CVI and children living with the condition. Participants were recruited via email lists for local and national organisations, local schools, specialist teachers and patient support groups who advertised the survey to professionals and families. We also invited those families who took part in the qualitative work to take part in the Delphi survey if they wished, but this was not expected or mandatory. Professionals who responded to flyers or email invitations were sent formal invitations to the survey with a link for completion online.

DelphiManager (a web-based Delphi survey data management system developed by COMET) was used to facilitate the construction, administration and analysis of the surveys. The first round of the survey presented the initial list of outcomes compiled using the literature review and interviews. Respondents were asked to rank the importance of measuring each outcome in CVI research using a 9-point scale, where 9 was “critical to include” and 1 was “not important at all”. We also invited respondents in the first round to add any additional outcomes that they felt should be included.

In the second round, respondents were shown the median ranking and range of rankings by each group, for each outcome and were reminded of their own ranking. They were given the opportunity to rank again items from the first round, and the additional items that had been suggested.

Stage 4: Consensus workshop

A workshop was held with attendees from both groups in Bristol, to ratify agreement on the most highly-ranked outcomes. We presented the three lists of outcomes: consensus ‘in’ with more than 70% from both groups ranking them as 7-9, consensus ‘out’ with more than 70% from both groups ranking them as 1-3, and partial or no agreement with less than 70% from either group ranking them as 7-9. Attendees discussed the inclusion or exclusion of the partial or no agreement list first, then moved on to discussing the rankings for all included outcomes and agreed a full list for the COS, after combining related outcomes to reduce overlap. Finally, attendees were asked to indicate their own personal “top 10” outcomes for inclusion. A shorter COS list was agreed containing all outcomes that at least one person selected for their personal top 10. With permission, the consensus meeting discussions were recorded.

The Protocol for the development of this COS is included in Supplementary Materials.

For peer review only - http://bmjopen.bmj.com/site/about/guidelines.xhtml

RESULTS

We followed our Protocol, but timing was later due to delays starting. For this reason we conducted 2 not 3 rounds of the Delphi survey.

Stage 1: Literature Review

5155 titles and abstracts were extracted from four databases. These were searched for evaluative studies and 22 were selected for full text review. Of these, 13 papers (10 studies) met the inclusion criteria and data were extracted. Three papers[11-13] reported on the use of bifocal spectacles for children with reduced accommodation and used visual outcomes (near vision, accommodative amplitude, preferred working distance) and educational outcomes (reading tests). Three papers [14-16]reported outcomes after treatments for symptomatic convergence insufficiency and they used visual (nearpoint of convergence, ability to fuse images, symptom score), educational (reading tests) and behavioural outcomes (child behaviour checklists). Two papers investigated the effects of treatments for children with reduced visual attention in the context of a diagnosed attentional disorder (ADHD): one used visual fields as the outcome[17] and one a score for visual attention[18]. Two papers investigated the effects of interventions to improve visuomotor skills: one involved prisms in children with hemispatial neglect and used visually-guided pointing as the outcome [19]and one used eye movement training and used eye movement, arms movement and ball-catching accuracy as outcomes[20]. The remaining papers investigated the effects of intraventricular stem cell transplant on visual behaviour[21]; music on attention to task[22] and a parenting programme on parent behaviour [23], all involving children with severe visual impairment. Thirty-seven individual outcomes were identified from the 13 papers in the review.

Stage 2: Qualitative interviews with families where a child has CVI

Families were from England, Scotland and Northern Ireland and one family lived in Eire. Eighteen interviews with parents/carers were completed. Six interviews with carried out with CYP with a parent also present. Eight parent/carer interviews took place by telephone, ten interviews were face-to-face.

Overall, children/young people's ages ranged from 3 to 17 years. Ten children were under the age of 11, 6 were between 11-16 years of age and one was aged 17 years. The six children who took part in separate interviews were aged 6-17 years (2 were in the younger group of 6-11 year olds and 4 were in the older group of 12-18 year olds). Of the six interviews conducted with children or young people, one used video call, one was by telephone and 4 were face to face. Other diagnoses were recorded as described by parents and included premature birth, cerebral palsy; chromosomal abnormalities; epilepsy; global developmental delay and autism.

From the analysis, 22 outcomes were identified across four themes of: (1) assessment and understanding of implications of CVI for CYP and families, (2) education, (3) family life, and (4) psychological wellbeing and quality of life.

All outcomes from the literature review and interviews were coded to the domains of the COMET taxonomy and these were used as section headings in the survey. We also included outcomes previously identified in a COS for children with neurodisability [24]. These were communication, emotional wellbeing, pain, sleep, mobility, self-care, independence, mental health, community and social life, behaviour, toileting and safety. Overlapping items were combined to reduce participant burden as much as possible. A final set of 23 discrete outcome domain items was included in the first round of the Delphi survey (Table 1). After discussion to combine similar outcomes and remove duplicate items, the 23 outcomes included in the first round included 5 relating to visual functions that came from the proportionate review of the literature, 8 outcomes relating to general health and abilities from the neurodisability COS and 10 outcomes from the results of the qualitative interviews.

For peer review only - http://bmjopen.bmj.com/site/about/guidelines.xhtml

Stage 3: Delphi Process

In total there were 126 participants in round 1 and 80 (64%) in round 2. These comprised QTVIs (29, then 21); Consultant Ophthalmologists (11 then 5); Consultant Paediatricians (4 then 2); Orthoptists (8 then 5); Habilitation Officers (8 then 5) Optometrists (3 then 2) and other professionals (17 then 12), plus the family members (46 then 28).

Twenty additional outcomes were suggested by participants in Round 1 of the survey, some from CYP, some from adult family members and some from professionals. These included 5 vision-related outcomes including sensitivity to light, ability to see colour and to pick out features in crowded scenes; other sensory outcomes related to hearing; more outcomes relating to social functioning and family wellbeing and one outcome related to adults around the child understanding CVI.

A total of 43 outcomes were therefore ranked in round 2. Table 1 shows the initial list of 23 outcomes included for ranking by participants as well as the 20 additional outcomes suggested by participants, that were ranked in round 2.

Table 1. Outcomes rated in Rounds 1 and 2 of the Delphi Survey of outcomes to include in a Core Outcome Set for Childhood CVI

Rounds	Outcome	Description given in survey	COMET taxonomy Domain
1&2	Ability to see things at a distance of at least 3 metres	For example looking at an eye chart with letters or pictures, with glasses if appropriate, visual acuity	Eye outcomes
1&2	Ability to focus on near things	For example toys, people's faces, letters or pictures, with glasses if appropriate	Eye outcomes
1&2	Ability to keep both eyes together on a near target	For example when reading, doing artwork, or using a computer or tablet	Eye outcomes
1&2	Ability to recognise visual targets	For example in picture books or on worksheets, adapted as necessary	Eye outcomes
1&2	Ability to track moving objects	For example follow the movements of other children, or animals or cars or balls	Eye outcomes
1&2	Mental health	For example, anxiety, depression, behavioural problems, self harm	Mental health including psychiatric problems
1&2	Child's Emotional wellbeing	For example child's level of confidence, happiness etc	Emotional functioning/wellbeing
1&2	Child's Self-perception	For example how child thinks of themselves, self-esteem	Emotional functioning/wellbeing
1&2	Sleep	Sleeping during the night or at appropriate times	General physiological/clinical
1&2	Tiredness/Fatigue	For example during the day, not just at night; lack of stamina, whatever the cause	General physiological/clinical
1&2	Mobility	For example child able to move around their physical environment, including physical obstacles, within the limits of any motor impairment or disability	Physical functioning
1&2	Self-care	For example washing, feeding and dressing, both practical and social aspects	Physical functioning
1&2	Independence	Able to go out and about and/or carry out activities of daily living appropriate to age and development, in and out of the home	Physical functioning
1&2	Safety	Awareness of danger (physical and social), appropriate to age or development, in and out of the home	Physical functioning

For peer review only - http://bmjopen.bmj.com/site/about/guidelines.xhtml

1&2	Toileting	As appropriate to physical abilities, including physical and social aspects	Physical functioning
1&2	Route-finding	Finding way around in the community and in both familiar and new settings e.g. school	Physical functioning
1&2	Communication	For example with family, friends and others; verbal and non-verbal	Social functioning
1&2	Community and social life	For example taking part in clubs, sport, parties, other leisure activities or entertainments	Social functioning
1&2	Behaviour	For example managing emotions at home, school and in the wider community, self regulation	Social functioning
1&2	Play	Ability to play, be part of playful activities, on own or with others	Social functioning
1&2	Learning	Whether at school or home-taught, learning and applying knowledge, attention and concentration	Cognitive functioning
1&2	Financial costs	For example costs incurred by parents; working time lost	Societal/carer burden
1&2	Family wellbeing	For example parental emotional well-being, family functioning	Societal/carer burden
2 only	Consistency of visual performance	For example, changes in ability to process visual information when tired, throughout the day	Eye outcome
2 only	Functional 3D skills	For example, making models in Lego, or craft modelling, doing jigsaw puzzles, dressing and feeding themselves, if appropriate	Eye outcomes
2 only	Ability to see colour		Eye outcomes
2 only	Sensitivity to light	For example, may need additional lighting or reduced lighting, or tinted glasses	Eye outcomes
2 only	Ability to deal with crowded or cluttered scene	For example, finding clothes, toys and people in cluttered or crowded spaces	Eye outcomes
2 only	Ability to watch TV, films or run computer games		Eye outcomes
2 only	Hearing	Being aware of sounds	General physiological/clinical
2 only	Auditory Understanding	For example, knowing the direction of sound	General physiological/clinical
2 only	Appreciation/enjoyment of music	Child enjoys listening to/experiencing music in whatever form	Social functioning
2 only	Pain	For example discomfort due to positioning, headaches or other sources of pain or discomfort	General physiological/clinical
2 only	Child's understanding of own needs and their condition	For example, knowing how CVI and other comorbidities affect them and what helps or hinders their daily lives, including being aware of visual field loss	Emotional functioning/wellbeing
2 only	Child's resilience	For example, child uses appropriate coping mechanisms or strategies to manage CVI and other conditions	Emotional functioning/wellbeing
2 only	Reading or accessing books		Cognitive functioning
2 only	Ability to cope with moving environment	For example, car travel, train travel, bus travel	Physical functioning
2 only	Environmental adaptations	Appropriate adaptations available including: learning materials, support with travel and transport	Physical functioning
2 only	Environmental adaptations are acceptable to the child	Child can or will access/use environmental adaptations as provided, for example, learning materials, support with travel and transport	Physical functioning

For peer review only - http://bmjopen.bmj.com/site/about/guidelines.xhtml

2 only	**Managing crowded environments**	**For example, in shops, cinemas, streets**	**Social functioning**
2 only	**Social life/relationships**	**For example, ability to make and sustain friendships and relationships**	**Social functioning**
2 only	**General Health**	**Self or proxy rated health, for example, 'how are you in yourself?'**	**General physiological/clinical**
2 only	**Relevant adults aware of CVI**	**Teachers, family etc know what CVI is and what helps the child**	**Social functioning**

Following the second round, the proportion of people ranking outcomes as of high importance with a ranking of 7-9 out of 9 was calculated. Of the total of 43 outcomes, 25 were “in” initially as >70% of each stakeholder group had ranked them as 7-9. Table 2 summarises the ranking scores and shows outcomes in red where both groups ranked them lower than 70%, in green where both ranked higher than 70% and in orange where one group ranked higher and one lower.

Table 2. Results of Delphi survey: percentages of 85 respondents who ranked the outcome with a score of 7-9 indicating they were important to include in the Core Outcome Set for Childhood CVI

Outcome	Domain	Families N = 30	Professionals N = 55
**Outcomes in first round**			
**Ability to see things at a distance of at least 3 metres**	Eye Outcomes	68%	67%
**Ability to focus on near things**	Eye Outcomes	87%	87%
**Ability to keep both eyes together on a near target**	Eye Outcomes	62%	53%
**Ability to recognise visual targets**	Eye Outcomes	97%	95%
**Ability to track moving objects**	Eye Outcomes	97%	76%
**Mental health**	Mental health	77%	83%
**Child's Emotional wellbeing**	Emotional functioning/wellbeing	83%	100%
**Child's Self-perception**	Emotional functioning/wellbeing	77%	87%
**Sleep**	General physiological/clinical	73%	76%
**Tiredness/Fatigue**	General physiological/clinical	87%	85%
**Mobility**	Physical functioning	93%	81%
**Self-care**	Physical functioning	80%	69%
**Independence**	Physical functioning	83%	89%
**Safety**	Physical functioning	97%	91%
**Toileting**	Physical functioning	60%	54%
**Route-finding**	Physical functioning	77%	65%
**Communication**	Social functioning	94%	96%
**Community and social life**	Social functioning	83%	87%
**Behaviour**	Social functioning	77%	72%
**Play**	Social functioning	80%	87%
**Learning**	Cognitive functioning	94%	88%
**Financial costs**	Societal/carer burden	53%	41%
**Family wellbeing**	Societal/carer burden	76%	90%
**Outcomes suggested by participants in Round 1**			
**Consistency of visual performance**	Eye Outcomes	80%	63%
**Functional 3D skills**	Eye Outcomes	78%	53%
**Ability to see colour**	Eye Outcomes	33%	26%
**Sensitivity to light**	Eye Outcomes	73%	63%
**Ability to deal with crowded or cluttered scene**	Eye Outcomes	83%	77%
**Ability to watch TV, films or run computer games**	Eye Outcomes	57%	39%
**Hearing**	General physiological/clinical	80%	68%
**Auditory Understanding**	General physiological/clinical	94%	68%

For peer review only - http://bmjopen.bmj.com/site/about/guidelines.xhtml

**Appreciation/enjoyment of music**	General physiological/clinical	50%	41%
**Pain**	General physiological/clinical	76%	72%
**Child's understanding of own needs and their condition**	Emotional functioning/wellbeing	64%	68%
**Child's resilience**	Emotional functioning/wellbeing	83%	81%
**Reading or accessing books**	Eye Outcomes	63%	54%
**Ability to cope with moving environment**	Eye Outcomes	94%	72%
**Environmental adaptations**	Physical functioning	87%	73%
**Environmental adaptations are acceptable to the child**	Physical functioning	89%	87%
**Managing crowded environments**	Social functioning	80%	63%
**Social life/relationships**	Social functioning	87%	90%
**General Health**	General physiological/clinical	73%	67%
**Relevant adults aware of CVI**	Delivery of care	94%	90%

Stage 4: Consensus workshop

Five people, two family representatives and three professionals, attended the consensus meeting in Bristol in March 2019. After a discussion, including of the 9 outcomes where there was uncertainty, some similar items were combined together and 28 outcomes were listed for inclusion in a final COS. Participants were then asked to vote on their ten most important outcomes. From this, a shorter COS list was produced, containing all the outcomes that at least one person put in their own top ten. The full list of all agreed outcomes is shown in Table 3. The top 15 of these comprise a short COS, of outcomes at least one person put in their personal "top ten".

Table 3. Results from Consensus meeting exercise: full list of items for inclusion in the Core Outcome Set for Childhood CVI and number of votes for participants' personal "top ten", at Consensus meeting

Number	Outcome	N who included outcome in their top ten (max 5)
1	Community/Social life and Relationships*	5
2	Learning and accessing books or screens*	5
3	Child emotional wellbeing	4
4	Tiredness/fatigue	4
5	Consistency of visual performance*	4
6	Managing in crowded environments*	4
7	Relevant adults aware of CVI*	4
8	Ability to deal with crowded or cluttered scene*	4
9	Mobility	3
10	Communication	3
11	Child's understanding of their own needs*	3
12	Safety	2
13	Environmental adaptations*	2
14	Ability to recognise visual targets	1
15	Family wellbeing	1
16	Ability to focus on near things	0
17	Ability to keep both eyes together on a near target	0
18	Ability to track moving objects	0
19	Child's Self-perception	0
20	Sleep	0

For peer review only - http://bmjopen.bmj.com/site/about/guidelines.xhtml

21	Self-care	0
22	Behaviour	0
23	Play	0
24	Functional 3D skills*	0
25	Sensitivity to light*	0
26	Ability to watch TV, films or run computer games*	0
27	Pain*	0
28	Ability to cope with moving environment*	0

There were 4 vision-related outcomes in the shorter COS (consistence of visual performance, ability to pick out features in a cluttered scene, ability to cope in a crowded environment and ability to recognise visual targets) as well as the outcome that adults around the child understood about CVI. In the short COS, 8 outcomes were suggested in part or completely, by the participants in round 1 (proposers included CYP, adult family members and professionals). Four were outcomes from the neurodisability COS. Two outcomes were voted as in their top ten by all 5 attendees: “community/interpersonal relations” and “ability to learn including accessing books or screens”.

Figure 2 summarises the different stages of the process to identify the COS and the results obtained.

DISCUSSION

This study brought together existing literature, families, professionals and us as researchers to reach a consensus on the most important outcomes to measure when conducting evaluative research into paediatric CVI. There was good engagement by the participants at all stages and, although the consensus meeting numbers were small, a clear plan for the day and a committed group of individuals were able to find common ground within their respective varied perspectives. Participants identified some outcomes with borderline results in Round 2 as important to include and did not reject any outcomes that had been included on the basis of Round 2 results. Although the participants were necessarily self-selecting and therefore not representative of the whole group or population with CVI, the consensus meeting validated, extended and ratified the Delphi survey results.

The COS of 28 items includes 12 vision-specific outcomes relating to a child’s visual abilities, supporting the value of a dedicated COS for children with brain-related vision impairments, as opposed t using a more general COS for children with neurodisability. Outcomes included being able to see targets in cluttered scenes, or when moving and consistency of visual performance. These aspects of visual performance are asked about in several questionnaires relating to CVI [25, 26] and the results of this exercise support the importance of asking about them when assessing a child with potential CVI. However, the majority of outcomes related to other domains in a child’s life and their ability to engage with other people and their surroundings. Several of the outcomes in the full COS were originally identified in the COS for children with neurodisability, reflecting the range of co-morbidities that are associated with CVI and were reported by our participants. The short COS featured 4 related to vision in real-world complex situations and the other 11 related to wellbeing, learning, interacting with the world and aspects of general health. This reflects the comments of the families in the interviews about the wide-ranging effects of CVI and indicates that professionals were also aware of the importance of non-visual outcomes.

This project was proportionate to the resources available and represents a first proposal of a COS for children with CVI, which is a relatively new field for clinical intervention studies. Our scope was broad and future research may be useful to identify more targeted COS for specific sub-groups of children with CVI or for specific situations. Many of the outcomes are themselves broad, multifaceted concepts such

For peer review only - http://bmjopen.bmj.com/site/about/guidelines.xhtml

as “Community, social life and relationships” and further work is needed to identify available validated measures for each outcome if available. However, the consistency with which these concepts were ranked as important indicates their importance to try and address, in some way. Increasing awareness of CVI was an important outcome for families and this may change as the condition becomes better understood.

Limitations of the study include that the literature review was specifically targeted to evaluations and some relevant studies may have been missed. We did not examine qualitative studies or health-related quality of life studies in children with CVI that may have elicited additional outcome domains. However, we included an established COS for children with neurodisability, plus multiple items derived from the interviews, and gathered additional suggestions from participants in the first round of survey, so are likely to have included most or all outcomes of key importance. We did not include users of core outcome sets such as researchers and journal editors, so did not capture their preferences in the consensus. We welcome discussion with colleagues to build on our proposed COS.

There were few children who were interviewed and/or who took part in the Delphi survey and these were necessarily those who could self-report to a reasonable level. We may have under-represented the views of younger or less able children - however all had been diagnosed with CVI and could speak to their experience about their lives. Only a small number of our participants were available to attend the consensus meeting which may have limited the range of opinions. Nevertheless all five participants were fully engaged and contributed to all aspects of the discussion and decision-making.

During the process there were repeated exercises to reduce overlapping or similar types of outcome. This was a subjective process and may have resulted in loss of some more specific outcomes that in other settings would be rated important. There is still a degree of overlap between the outcomes and another group may have refined them differently. However, there was strong agreement that both visual function related outcomes and child-centred outcomes relating to life chances and quality of life, were of crucial importance to include.

A strength of this work is the high level of engagement of participants, the inclusion of children as well as parents and the broad levels of agreement between families and professionals. We used a variety of ways to identify relevant candidate outcomes and followed the COMET process that has been optimised iteratively over time. Several of the outcomes in the full COS and the short COS were suggested by the participants during the process. This indicates the value of inviting suggestions from participants to gain perspectives not suggested by the literature review or the interviews, perhaps because the context of rating outcomes stimulated different ideas from the more general discussion topics in the interviews.

Conclusions

A first COS for childhood CVI has been proposed. More work is needed to identify which tools might best be used to measure these and some may have to be addressed qualitatively if no validated tools exist. Future COS research could also examine modifications of the full COS for more specified groups or interventions, i.e. with a more restricted scope, or any changes that might result from including COS users e.g. journal editors or whether a having larger number of attendees at a consensus meeting leads to different selections of outcomes. Future research into interventions to help children with CVI should include if possible, at least the short COS items, to increase relevance for the research for families and professional and to aid cross-study comparisons.

For peer review only - http://bmjopen.bmj.com/site/about/guidelines.xhtml

Acknowledgements

We are very grateful to the families and professionals who took part in this study, including the attendees of our consensus meeting: Kirsty Goodman, Janet Harwood, Kate Murton, Grainne Ni Dhuill, and Penny Warnes. We are also grateful to the members of the PenCRU Family Faculty who advised.

Author Contributions

The study was designed and led by CW, with hep from all authors. CM advised on all aspects, CB did the literature search, TG led on the participant interviews and RW, TG and AP led on the Delphi study. AP drafted the first version of the manuscript and all authors contributed to revisions and approved the final manuscript.

Funding

This research was funded by the National Institutes of Health Research (NIHR) as part of a Senior Research Fellowship for CW: SRF_2015_08_005.

Disclaimer

The views expressed in this publication are those of the author(s) and not necessarily those of the NIHR, NHS or the UK Department of Health and Social Care.

Competing Interests

The authors have no competing interests.

Data Sharing

Anonymised data will be shared on reasonable request.

For peer review only

Legend for Table 2:
Green= 70% or more of each group rated outcome as 7-9, so put forward for inclusion
Orange = 70% or more of one group only rated outcome as 7-9, so put forward for discussion at Consensus meeting
Red= fewer than 70% of respondents in each group rated the outcome as 7-9, so outcome not put forward for inclusion

Legend for Table 3:
Shaded rows are outcomes suggested in full or in part by respondents in the Delphi survey

REFERENCES

1. Sakki, H.E.A., et al., *Is there consensus in defining childhood cerebral visual impairment? A systematic review of terminology and definitions.* Br J Ophthalmol, 2018. **102**(4): p. 424-432.
2. Williamson, P.R., et al., *Developing core outcome sets for clinical trials: issues to consider.* Trials, 2012. **13**: p. 132.
3. Kirkham, J.J., et al., *Core Outcome Set-STAndards for Development: The COS-STAD recommendations.* PLoS Med, 2017. **14**(11): p. e1002447.
4. Kirkham, J.J., et al., *Core Outcome Set-STAndards for Reporting: The COS-STAR Statement.* PLoS Med, 2016. **13**(10): p. e1002148.
5. Williamson, P.R., et al., *The COMET Handbook: version 1.0.* Trials, 2017. **18**(Suppl 3): p. 280.
6. Hasson, F., S. Keeney, and H. McKenna, *Research guidelines for the Delphi survey technique.* J Adv Nurs, 2000. **32**(4): p. 1008-15.
7. Rowe, F.J., L.R. Hepworth, and J.J. Kirkham, *Development of core outcome sets for vision screening and assessment in stroke: a Delphi and consensus study.* BMJ Open, 2019. **9**(9): p. e029578.
8. Staniszewska, S., et al., *GRIPP2 reporting checklists: tools to improve reporting of patient and public involvement in research.* BMJ, 2017. **358**: p. j3453.
9. Williams, C., et al., *How to help children with neurodevelopmental and visual problems: a scoping review.* Br J Ophthalmol, 2014. **98**(1): p. 6-12.
10. Dodd, S., et al., *A taxonomy has been developed for outcomes in medical research to help improve knowledge discovery.* Journal of Clinical Epidemiology, 2018. **96**: p. 84-92.
11. Nandakumar, K., et al., *Bifocals in Down syndrome study (BiDS): analysis of video recorded sessions of literacy and visual perceptual skills.* Clin Exp Optom, 2011. **94**(6): p. 575-85.
12. Nandakumar, K. and S.J. Leat, *Bifocals in children with Down syndrome (BiDS) - visual acuity, accommodation and early literacy skills.* Acta Ophthalmol, 2010. **88**(6): p. e196-204.
13. Stewart, R., J. Woodhouse, and L.D. Trojanowska, *In focus: the use of bifocal spectacles with children with Down's syndrome.* Ophthalmic and Physiological Optics, 2005. **25**: p. 514-522.
14. Borsting, G., et al., *Behavioral and Emotional Problems Associated With Convergence Insufficiency in Children: An Open Trial.* Journal of Attention Disorders, 2016. **20**: p. 836-844.
15. Convergence Insufficiency Treatment Trial Study, G., *Randomized clinical trial of treatments for symptomatic convergence insufficiency in children.* Arch Ophthalmol, 2008. **126**(10): p. 1336-49.
16. Scheiman, M., et al., *Effect of treatment of symptomatic convergence insufficiency on reading in children: a pilot study.* Clin Exp Optom, 2018. **101**(4): p. 585-593.
17. Martin, L., et al., *Visual fields in children with attention-deficit / hyperactivity disorder before and after treatment with stimulants.* Acta Ophthalmol, 2008. **86**(3): p. 259-64.

For peer review only

For peer review only - http://bmjopen.bmj.com/site/about/guidelines.xhtml

18. Vaisman, N., et al., *Correlation between changes in blood fatty acid composition and visual sustained attention performance in children with inattention: effect of dietary n-3 fatty acids containing phospholipids.* Am J Clin Nutr, 2008. **87**(5): p. 1170-80.
19. Riquelme, I., et al., *Use of prism adaptation in children with unilateral brain lesion: Is it feasible?* Res Dev Disabil, 2015. **43-44**: p. 61-71.
20. Miles, C.A., et al., *Quiet eye training aids the long-term learning of throwing and catching in children: Preliminary evidence for a predictive control strategy.* Eur J Sport Sci, 2017. **17**(1): p. 100-108.
21. Luan, Z., et al., *Neural stem/progenitor cell transplantation for cortical visual impairment in neonatal brain injured patients.* Cell Transplant, 2013. **22 Suppl 1**: p. S101-12.
22. Robb, S.L., *Music interventions and group participation skills of preschoolers with visual impairments: raising questions about music, arousal, and attention.* J Music Ther, 2003. **40**(4): p. 266-82.
23. Overbeek, M.M., et al., *The effectiveness of VIPP-V parenting training for parents of young children with a visual or visual-and-intellectual disability: study protocol of a multicenter randomized controlled trial.* Trials, 2015. **16**: p. 401.
24. Morris, C., et al., *Meaningful health outcomes for paediatric neurodisability: Stakeholder prioritisation and appropriateness of patient reported outcome measures.* Health Qual Life Outcomes, 2015. **13**: p. 87.
25. University of Ulster. *CVI Resources*. Available from: https://www.ulster.ac.uk/research/topic/biomedical-sciences/research/optometry-and-vision-science-research-group/vision-resources/professionals/cerebral-visual-impairment-assessment.
26. Ortibus, E., et al., *Screening for cerebral visual impairment: value of a CVI questionnaire.* Neuropediatrics, 2011. **42**(4): p. 138-47.

For peer review only

Figure 1. Box describing Patient and public involvement in Core Outcome Set for childhood CVI, using GRIPP2 headings

How was the development of the research question and outcome measures informed by patients' priorities, experience, and preferences?
Parents and patients told us about the wide-ranging spectrum of effects that they felt CVI had on their children's lives. The emphasised the importance of poor mental health for children with CVI but no support.

How did you involve patients in the design of this study?
Parents approved the approach recommended by the Comet Initiative and recommended including home-schooled children if possible.

Were patients involved in the recruitment to and conduct of the study?
Parents recruited interviewees and Delphi participants via word-of-mouth using a closed Facebook group and parent networks. Parents recommended ways to engage with child interviewees. Parents and children reviewed and revised the wording of the Delphi surveys.

How will the results be disseminated to study participants?
We will provide a summary of the process and the findings from the qualitative interviews, Delphi process and consensus meeting to all participants. We will present the findings to the yearly meeting of a parent support group (www.cvisociety), post a summary on our study website, present the findings at conferences and submit a paper to a peer reviewed journal.

For peer review only

Figure 2

Flowchart of development of Core Outcome set for Childhood CVI

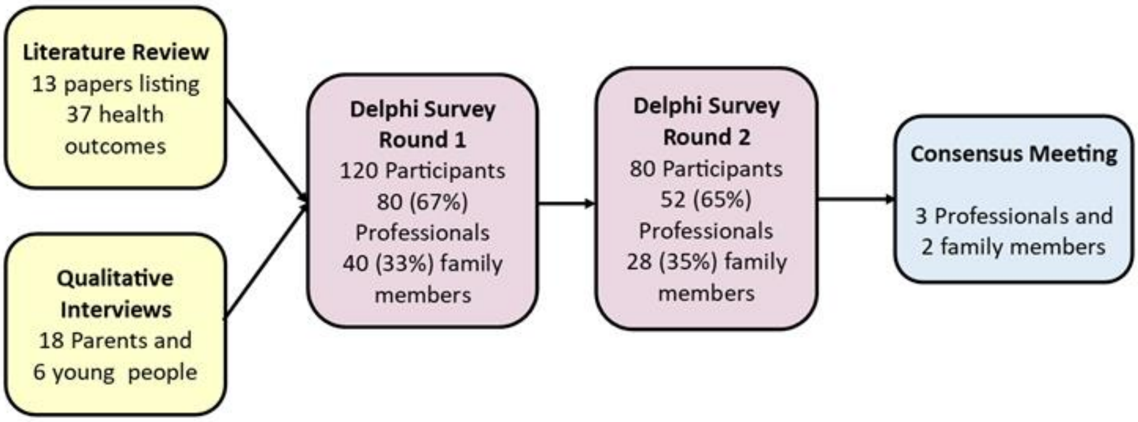

review only

University of BRISTOL

Research Protocol

The CVI Project: A Core Outcome Set for children with Cerebral Visual impairments (COS-CVI)

NHS National Institute for Health Research

Cathy Williams & Anna Pease

Contents

Project Team 3
A Core Outcome Set for Children with Cerebral Vision Impairments (COS-CVI) 4
Background 5
Methods 6
Scope 6
Public Involvement 6
Phase 1. Identifying Candidate Outcomes 6
Proportionate Systematic Review 6
Qualitative work 7
Phase 2. Rating the importance of Candidate Outcomes using a Delphi Survey 9
Panels 9
Software, nature and ordering of questions 9
Number of rounds and nature of feedback given 9
Consensus workshop 10
Discussion and implementation 11
Gantt Chart for Core Outcome Set Development 12
Figure 1: Core Outcome Set Development Flowchart 13
References 14

For peer review only

Project Team

Ms Cathy Williams
Post: Reader in Paediatric Ophthalmology
Email: Cathy.Williams@bristol.ac.uk
Dept: Centre for Child and Adolescent Health, School of Social and Community Medicine
Address: University of Bristol, Oakfield House, Oakfield Grove, Bristol, BS8 2BN
Tel: 0117 3313388

Dr Anna Pease
Post: Senior Research Associate
Email: A.pease@bristol.ac.uk
Dept: Centre for Child and Adolescent Health, School of Social and Community Medicine
Official Address: University of Bristol, Oakfield House, Oakfield Grove, Bristol, BS8 2BN
Tel: 0117 331 3310

Dr Trudy Goodenough
Post: Senior Research Associate
Email: Trudy.Goodenough@bristol.ac.uk
Dept: Centre for Child and Adolescent Health, School of Social and Community Medicine
Official Address: University of Bristol, Oakfield House, Oakfield Grove, Bristol, BS8 2BN
Tel: 0117 331 3310

Ms Rose Watanabe
Post: Executive Assistant
Email: rose.watanabe@bristol.ac.uk
Dept: Centre for Child and Adolescent Health, School of Social and Community Medicine
Official Address: University of Bristol, Oakfield House, Oakfield Grove, Bristol, BS8 2BN
Tel: 0117 331 4005

Dr Christopher Morris
Post: Senior Research Fellow
Email: Christopher.Morris@exeter.ac.uk
Dept: Child Health Group Peninsula Cerebra Research Unit for Childhood Disability Research (PenCRU)
Official Addess: University of Exeter Medical School, South Cloisters, St Luke's Campus, Exeter, EX1 2LU
Tel: 01392 262980

Ms Cath Borwick
Post: Research Engagement Librarian - Medical Faculties
Email: cath.borwick@bristol.ac.uk
Dept: Medical Library
Official Address: Medical Sciences Building, University of Bristol, Bristol BS8 1TD
Tel: (0117) 331 1504 or 394 1079

Funding Source: National Institute of Health Research (NIHR) Senior Research Fellowship Award

For peer review only - http://bmjopen.bmj.com/site/about/guidelines.xhtml

For peer review only

A Core Outcome Set for Children with Cerebral Vision Impairments (COS-CVI)

Abstract

In the current climate of financial challenges and limited resources, the importance of best use of available funds is paramount. Core outcome sets are condition-specific sets of outcomes that are developed by and represent the concerns of a range of stakeholders including patients, carers, professionals and policy makers. The aim of this project is to develop a COS for use in evaluative research on interventions that aim to improve outcomes for children with brain-related vision impairments, commonly known as cerebral visual impairments or CVI.

Methods

This project is nested within a 5-year programme of work investigating the prevalence, impact and effectiveness of support for primary school age children with CVI. We will use methods recommended by the COMET Initiative (Core Outcome Measures in Effectiveness Trials) including a proportionate review of what's been measured before; qualitative work to bring in the voices of children and families; a Delphi survey involving parents, children, professionals in medicine and teaching and therapists and a consensus meeting to determine the most important outcomes. We will then review the available tools and decide how best to measure the selected outcomes.

Discussion and Implementation

Our methods are proportionate to the resources available in the 5-year programme and we will reflect and discuss any consequent limitations of this on our findings. We will then use the COS and the tools that best measure the selected outcomes in a feasibility cluster-randomised controlled trial (fcRCT). Outcomes from the fcRCT will include the acceptability and practicality of the COS and these may be useful for further development of the COS.

For peer review only - http://bmjopen.bmj.com/site/about/guidelines.xhtml

Background

Brain-related vision impairments, commonly known as cerebral visual impairments or CVI, are frequently reported in studies of patients who have suffered a form of injury to their central nervous syndrome or who are born with an abnormality in the brain structure or function.

CVI is an umbrella term used by many to include abnormalities in visual acuity, or contrast sensitivity or colour; ocular motility; visual field and the conscious and unconscious filtering or processing of visual input (1). However, in the current World Health Organisation (WHO) classification of diseases ICD10, only codes for acuity and field loss are used in the description of "visual impairment (VI)" and consequently in many countries, including the UK, certification of VI involves only those visual functions (2). There is not as yet a consensus within the UK or internationally as to how other impairments of visual functioning, such as eye movements or vision processing, should be characterised. The new ICD classification ICD11 is likely to contain addition codes for complex vision impairment affecting functions such as reading or navigation and this may aid future research and resource allocation (3). In the meantime, there are very few studies to guide parents or vision professionals or therapists on how much of a person's difficulties might be attributable to CVI, nor on how best to help them.

Individuals most at risk of CVI are those with childhood-onset neurodevelopmental disorders (1), and adults who develop neurological disorders particularly including stroke and dementia (4). Some of the problems relating to having CVI are likely to be present at all ages. Children with CVI have additional specific needs and problems relating to their development from infancy to adulthood and their need for education whilst adults who acquire their neurological problems later in life may have different problems relating to employment and adjustment to their new impairment.

Currently there are 17 projects registered in the "Eyes and Vision" section of the COMET database (5). All but two of them focus on adults rather than children. One of the adult projects is however specifically on CVI after stroke and will be contacted as there may be overlap between child and adult outcomes after CVI.

As part of establishing to what extent children with CVI might be helped by interventions in a future RCT, we will therefore develop a specific COS for children with CVI. We will take an inclusive approach and include CVI however diagnosed, to reflect (a) the current lack of consensus about what brain related impairments should be regarded as visual and (b) variation in availability of detailed vision testing to elicit all the potential manifestations of CVI.

For peer review only

Methods

We will register the study with the COMET Initiative. We will seek ethical committee permission through the University Faculty Research Ethics Committee (FREC).

Scope

We will include children of school-age up to 18 as they will be able to contribute their perspectives relating to their own recent past as well as their present, although the future fcRCT will be focussing on primary school children. We will consider outcomes of interventions to help children with CVI in any setting, including evaluative trials and routine practice in hospitals and schools. Our primary aim however is to arrive at an agreed COS for use in the planned fcRCT. We will use the PICO format to specify the scope of the study. The scope is participants in the UK and Ireland, though broader generalizability internationally could be explored subsequently.

Public Involvement

We already have had input from a local group of families whose children have CVI and from the PenCRU "Family Faculty" coordinated by University of Exeter. These are a group of parents whose children have a range of neurodevelopmental conditions. We will ask their advice on practical details of interacting with children with neurodevelopmental conditions in the planned qualitative interviews. They will also be consulted on interpreting the results and disseminating the findings.

Phase 1. Identifying Candidate Outcomes

Proportionate Systematic Review

We will review the literature to see which outcomes have been measured in interventions to help children with CVI.

Search strategy
We will include directly assessed, patient-reported and proxy-reported outcomes for children with CVI according to any definition made by a relevant professional (eg a vision specialist, a neuropsychologist or a doctor.

Types of Studies
Any interventional study that aims to improve outcomes for children with CVI as defined above

Types of Participants
Children up to 18 with CVI as defined above

For peer review only - http://bmjopen.bmj.com/site/about/guidelines.xhtml

Inclusion criteria

We will include studies that report on children with what the study reports as CVI, however defined. We will include experimental studies and observation studies that describe associations with CVI defined by a relevant professional

Exclusion criteria

We will exclude studies where it is not possible to separate adult from child data, where there is no description of the visual behaviour used to define the child as having CVI and studies not in English.

Data extraction

We will record each type of outcome recorded in included papers and the instrument used to measure or ascertain the outcome. We will record study type and participant characteristics as well as numbers of respondents and non-respondents, or non-applicable responses, for each outcome. Data will be extracted by two reviewers and compared and consensus reached by discussion with a third reviewer.

Synthesis

We will list all outcomes from the papers in a database as well as the instruments used. We will code and organize them in two ways. Firstly, we will use the forthcoming taxonomy proposed by the COMET Initiative (6). Secondly, we will map them to the domains in the WHO International Classification of Functioning (ICF) insofar that is possible. These two models will be used to sort the outcomes as per Wilson and Cleary (7) into biological or functional measures; activity or participation measures, mental health states and quality of life.

We will submit draft coding and lists to our advisory panels for discussion and refinement.

Qualitative work

Aim

The aim of the qualitative work will be to "fill in the gaps" revealed by the systematic review and capture the language and perspectives of children and their families.

Participants

Participants will be children and young people aged 6 - 18 years with a diagnosis of CVI, using any definition, and/or parents or carers of children with this diagnosis.

For peer review only

Sampling method

To include a range of ages and physical capabilities, maximum variation purposive sample will be used to recruit families to a matrix including younger (6-11 years old) and older (12-18 years old) children, and those with and without a diagnosis of cerebral palsy.

Recruitment

We will recruit children and young people from three sources initially and will use a snowballing approach to recruit more if needed. Firstly, we will ask the local Specialist teachers for Vision Impairment to post a notice with information and contact details on their website. They will also notify individuals they think might be interested. We will visit a local school for children with visual impairment (The WESC Foundation, Specialist Centre for Visual Impairment, Exeter) and distribute notices and information. Thirdly we will give the same information to a national parent support group for families of children with CVI (www.cvisociety.org.uk).

Data collection – interviews

Interviews will be carried out in person, at school or at home as preferred or as convenient. Children will be interviewed with a trusted adult present (this can be a parent or member of school staff), after their parent or carer has been interviewed. All interviews will be audio-recorded with consent. A topic guide based on the systematic review and comments from the advisory groups and based on approaches already reported and successful with similar-aged children will be used (for example an activity discussing “a day in the life of a child with CVI”). Interviews will be expected to take 30-45 minutes for adults and 10-30 minutes for children. When interviewing children a range of creative activities will help us to elicit views of young people about what matters most to them. We will use drawings, stories and a tablet to support these approaches to follow the topic guide.

Interviews will continue with families until the point of diminishing returns, when little or no new perspectives are forthcoming and no new outcome domains are being identified. We expect to conduct interviews with approximately 30 families in total.

Analysis

A framework analysis consisting of five steps will be used. First familiarisation with the data will allow for submersion in the topic area. Secondly an initial framework will be applied by coding key issues in the transcripts. Third, the codes will be indexed into categories, fourth, thematic charts will be produced and compared by independent coders (members of the research team) and finally the charts will be interpreted into themes using key ideas and quotes from the original data.

For peer review only - http://bmjopen.bmj.com/site/about/guidelines.xhtml

Mapping to a framework

We will map the data obtained from the qualitative interviews into categories according to the two coding schemes (the COMET taxonomy and the ICF) described above.

Phase 2. Rating the importance of Candidate Outcomes using a Delphi Survey

We will carry out a Delphi survey to rate the importance of the outcomes assembled from the systematic review and the interview data. A Delphi survey is an iterative process of defining and refining importance of outcomes, using several rounds of surveys involving parents, children, professionals in medicine and teaching and therapists. The process ends with a consensus meeting to determine the most important outcomes.

Panels

We will invite individuals to participate in either of two panels:
1. Professionals (ophthalmologists, optometrists, orthoptists, paediatricians, therapists, teachers)
2. Parents and YP and children.

We will ask local and national organisations to advertise the survey to professionals. We will ask local schools, the Specialist teachers and patient support groups to advertise the survey to families including children themselves if they want to participate, potentially with the support of a parent or carer, or independently. We will also invite those families who have taken part in the qualitative work to take part in the Delphi if they wish, but this is not expected or mandatory. We will invite individuals willing to participate to register through an online system (COMET's Delphi Manager) or to contact the study team directly. We will record the professional role, years in role, institution and area of the country for professionals taking part. We will record age, ethnicity and diagnosis/es of affected children for the families taking part.

Software, nature and ordering of questions

We will use DelphiManager (a web based data management system developed by COMET) to facilitate the construction and delivery of our surveys. The software allows data to be extracted and consolidated at every round, as well as providing analysis of responses.

Number of rounds and nature of feedback given

For peer review only - http://bmjopen.bmj.com/site/about/guidelines.xhtml

Round 1: In the initial stage of the survey we will present the findings of the systematic review and we will ask respondents to rate the importance of each using a 9 point scale in which 1-3 relates to “less important” and 7-9 relates to very important. We will also ask respondents to add any additional outcomes that they feel should be included. Those items that score a median of 7 or above and/or 70% from either panel rating the outcome as 7-9 will be carried into round 2.

Round 2: Following the initial stage of rating the systematic review findings and generating further outcomes, we will add in the list of outcomes provided through the qualitative work (taking out any overlapping items from each stream) and present the complete list in Round 2. We will ask respondents to rate each item as before. For those items that have been rated previously, respondents will be shown the median scores and range for three groups; (1) the scores from professionals, (2) the scores from families and (3) the overall median score from both panels. Those items that score a median of 7 or above and/or 70% from either panel rating the outcome as 7-9 will be carried into round 3.

Round 3: In round 3, respondents will be shown their score from Round 2 as well as the median score and range from each panel who responded for each item. We will include items where there is agreement between panels that items are important and also items where there is polarisation (e.g. professionals scored high but families scored low) between panels and indicate this in the survey. Space will be provided for respondents to add their own comments on how they have rated items and to add any further considerations.

We will send each final survey for each round to the Faculty of Health Sciences Research Ethics Committee for information.

Consensus workshop

We will hold a consensus workshop with attendees from both panels in Bristol, to ratify agreement of the more important outcomes. We will highlight that the core outcomes are a minimum set for measuring change and that interventions may add in other outcomes as they see fit. We will present a list of outcomes identified in round 3 with a median score of 7 or above with 70% from either panel rating the outcome as 7-9. Research team members will facilitate the workshop. All UK participants will be invited to attend. Attendees will be shown the list of outcomes to be ratified prior to the workshop. The workshop will comprise three parts.

For peer review only - http://bmjopen.bmj.com/site/about/guidelines.xhtml

Part 1 will include introductions for participants and introduce the format for the workshop. This will include the aims of the day and the threshold for inclusion of items into the final core set.
Part 2 will see participants discuss and rate the importance of items in mixed stakeholder groups, with a facilitator for each group.
Part 3 will bring the participants back together to arrange the final set of outcomes into importance and will be asked to endorse the proposed final set.

Discussion and implementation

Once we have identified what to include in the fcRCT from the above process, we will review the most feasible way to collect data on these outcomes. This COS project is necessarily proportionate to the resources in the NIHR Fellowship award that funds it and additional work such as the development of new assessment tools, is outside the scope and resources available. We will use the COS obtained from the consensus meeting to inform selection of outcome measures in the fcRCT. We will collect data on the feasibility and acceptability of the data collection for these outcome measures and use this to guide any further developments of a COS for children with CVI.

Timescale

The Gantt chart on page 12 indicates the sequence of events and the originally planned timescale. The qualitative work will extend into July due to staff changes and a

For peer review only

Gantt Chart for Core Outcome Set Development

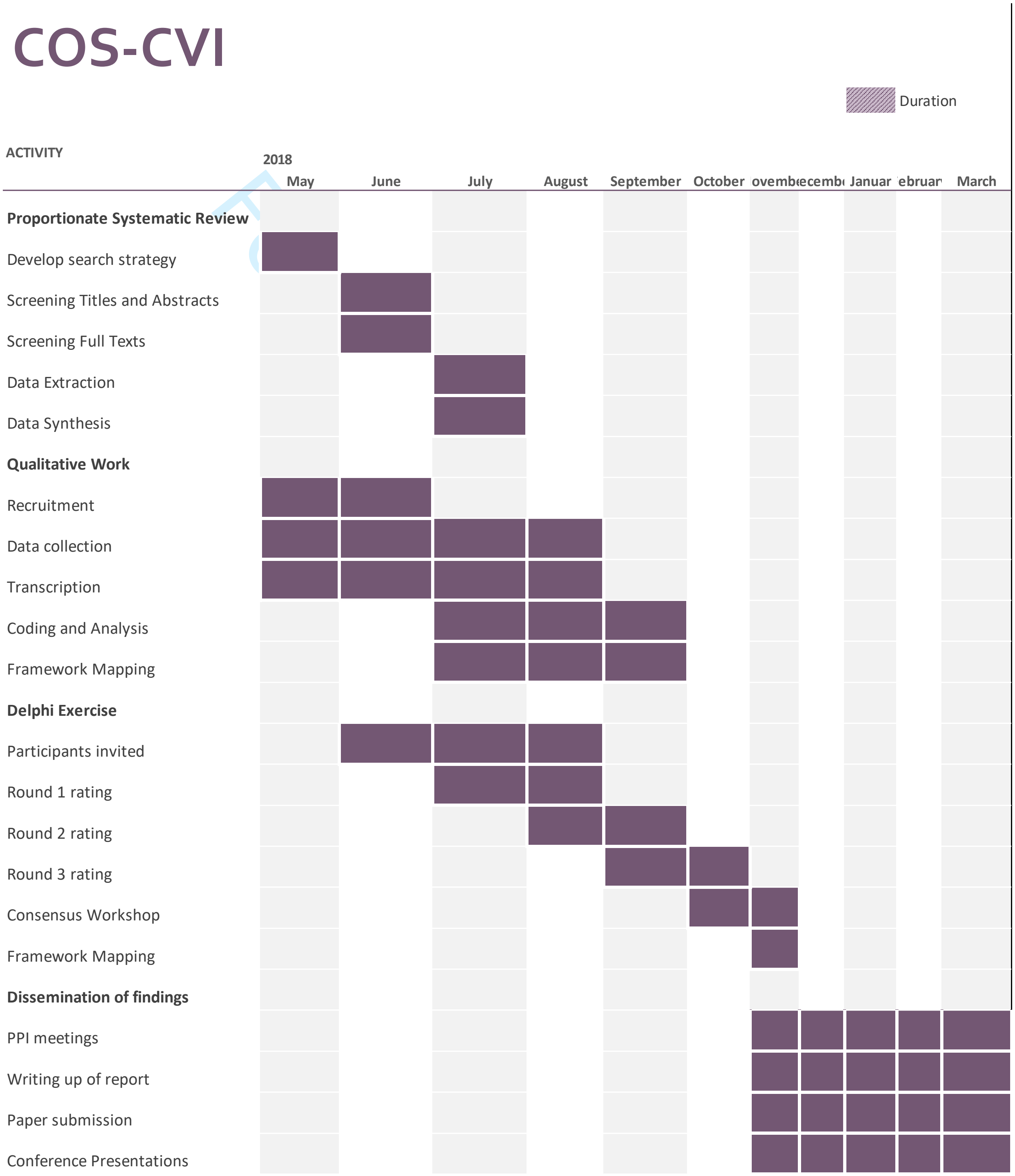

For peer review only - http://bmjopen.bmj.com/site/about/guidelines.xhtml

Figure 1: Core Outcome Set Development Flowchart

Phase 1: Proportionate Systematic Review

- Identify outcomes in RCTs involving children with CVI

Phase 2: Qualitative Interviews

- Capture perspectives and language of children with CVI and their families

Phase 3: Delphi Exercise

- Rate the importance of candidate outcomes
- Two panels - professionals and families

Round 1

1. Present outcomes from systematic review and rate on likert scale from 1 to 9
2. Provide feedback on ratings (optional)
3. Suggest new outcomes

Include Outcomes which score a median of 7 or above and/or 70% from either panel rating the

Round 2

1. Present outcomes from round 1 and qualitative interviews
2. Participants see own score, median for each panel and overall median score
3. Re-rate items from 1-9
4. Provide feedback on ratings (optional)

Include Outcomes which score a median of 7 or above and/or 70% from either panel rating the

Round 3

1. Present outcomes from round 2
2. Participants see own score, median for each panel and overall median score
3. Re-rate items from 1-9
4. Provide feedback on ratings (optional)

Consensus Workshop

1. Introductions and aims for the workshop
2. Break out groups to discuss and rate items
3. Consensus reached on core outcome set

Dissemination and Implementation

1. Report writing
2. Data collection methods for core outcomes reviewed
3. Select outcome measures for future fcRCT

Fig 1. Core Outcome Development Flowchart. Adapted from Tong et al. (2016)

For peer review only

References

1. Philip, S. S. and Dutton, G. N. (2014), Identifying and characterising cerebral visual impairment in children: a review. Clin Exp Optom, 97: 196–208. doi:10.1111/cxo.12155

2. https://www.gov.uk/government/publications/guidance-published-on-registering-a-vision-impairment-as-a-disability

3. https://icd.who.int/dev11/proposals/

4. Visual Perceptual Consequences of Stroke Fiona Rowe & VIS Group UK. Strabismus Vol. 17, 1,2009

5. http://www.comet-initiative.org

6. Trials. 2017 Jun 20;18(Suppl 3):280. doi: 10.1186/s13063-017-1978-4. The COMET Handbook: version 1.0.

7. Wilson IB, Cleary PD: Linking clinical variables with health-related quality of life: a conceptual model of patient outcomes. JAMA 1995, 273(1):59-65.

For peer review only

SECTION/TOPIC	ITEM No.	CHECKLIST ITEM	
**TITLE/ABSTRACT**			
Title	1a	Identify in the title that the paper reports the development of a COS	✓
Abstract	1b	Provide a structured summary	✓
**INTRODUCTION**			
Background and Objectives	2a	Describe the background and explain the rationale for developing the COS.	p4
	2b	Describe the specific objectives with reference to developing a COS.	p4
Scope	3a	Describe the health condition(s) and population(s) covered by the COS	p4
	3b	Describe the intervention(s) covered by the COS.	p4
	3c	Describe the setting(s) in which the COS is to be applied.	p4
**METHODS**			
Protocol/Registry Entry	4	Indicate where the COS development protocol can be accessed, if available, and/or the study registration details.	Supp
Participants	5	Describe the rationale for stakeholder groups involved in the COS development process, eligibility criteria for participants from each group, and a description of how the individuals involved were identified.	p5
Information Sources	6a	Describe the information sources used to identify an initial list of outcomes.	p5
	6b	Describe how outcomes were dropped/combined, with reasons (if applicable).	p6
Consensus Process	7	Describe how the consensus process was undertaken.	p6
Outcome Scoring	8	Describe how outcomes were scored and how scores were summarised.	p6
Consensus Definition	9a	Describe the consensus definition.	p6
	9b	Describe the procedure for determining how outcomes were included or excluded from consideration during the consensus process.	p6
Ethics and Consent	10	Provide a statement regarding the ethics and consent issues for the study.	p4
**RESULTS**			
Protocol Deviations	11	Describe any changes from the protocol (if applicable), with reasons, and describe what impact these changes have on the results.	p7
Participants	12	Present data on the number and relevant characteristics of the people involved at all stages of COS development.	p7-8
Outcomes	13a	List all outcomes considered at the start of the consensus process.	Table2
	13b	Describe any new outcomes introduced and any outcomes dropped, with reasons, during the consensus process.	Table 2
COS	14	List the outcomes in the final COS.	Table3
**DISCUSSION**			
Limitations	15	Discuss any limitations in the COS development process.	p13
Conclusions	16	Provide an interpretation of the final COS in the context of other evidence, and implications for future research.	p13
**OTHER INFORMATION**			
Funding	17	Describe sources of funding/role of funders.	p14
Conflicts of Interest	18	Describe any conflicts of interest within the study team and how these were managed.	p14, p11

doi:10.1371/journal.pmed.1002148.t001

1467x1100mm (72 x 72 DPI)

Development of a core outcome set for evaluative research into Paediatric Cerebral Visual Impairment (CVI), in the UK and Eire

Journal:	*BMJ Open*
Manuscript ID	bmjopen-2021-051014.R1
Article Type:	Original research
Date Submitted by the Author:	25-Aug-2021

Complete List of Authors:	Pease, Anna; University of Bristol,<br>Goodenough, Trudy; University of Bristol, Bristol Medical School<br>Borwick, Cath; University of Bristol<br>Watanabe, Rose; University of Bristol<br>Morris, Christopher; University of Exeter Medical School<br>Williams, Cathy; University of Bristol

**Primary Subject Heading**:	Ophthalmology
Secondary Subject Heading:	Paediatrics
Keywords:	Paediatric ophthalmology < OPHTHALMOLOGY, EPIDEMIOLOGY, QUALITATIVE RESEARCH, Paediatric neurology < NEUROLOGY

SCHOLARONE™
Manuscripts

BMJ

I, the Submitting Author has the right to grant and does grant on behalf of all authors of the Work (as defined in the below author licence), an exclusive licence and/or a non-exclusive licence for contributions from authors who are: i) UK Crown employees; ii) where BMJ has agreed a CC-BY licence shall apply, and/or iii) in accordance with the terms applicable for US Federal Government officers or employees acting as part of their official duties; on a worldwide, perpetual, irrevocable, royalty-free basis to BMJ Publishing Group Ltd ("BMJ") its licensees and where the relevant Journal is co-owned by BMJ to the co-owners of the Journal, to publish the Work in this journal and any other BMJ products and to exploit all rights, as set out in our licence.

The Submitting Author accepts and understands that any supply made under these terms is made by BMJ to the Submitting Author unless you are acting as an employee on behalf of your employer or a postgraduate student of an affiliated institution which is paying any applicable article publishing charge ("APC") for Open Access articles. Where the Submitting Author wishes to make the Work available on an Open Access basis (and intends to pay the relevant APC), the terms of reuse of such Open Access shall be governed by a Creative Commons licence – details of these licences and which Creative Commons licence will apply to this Work are set out in our licence referred to above.

Other than as permitted in any relevant BMJ Author's Self Archiving Policies, I confirm this Work has not been accepted for publication elsewhere, is not being considered for publication elsewhere and does not duplicate material already published. I confirm all authors consent to publication of this Work and authorise the granting of this licence.

For peer review only

Development of a core outcome set for evaluative research into Paediatric Cerebral Visual Impairment (CVI), in the UK and Eire

Pease, A[1], Goodenough, T[1], Borwick, C[1], Watanabe, R[1], Morris, C[2], and Williams, C[1],

1.Bristol Medical School, 1-5 Whiteladies Road, University of Bristol, Bristol BS8 1NU, UK

2.PenCRU (Peninsula Childhood Disability Research Unit), University of Exeter Medical School, University of University, UK

Corresponding Author

C Williams
1-5 Whiteladies Road
Clifton
Bristol
BS8 1NU
Cathy.Williams@bristol.ac.uk

Main text words =

For peer review only

ABSTRACT

Objectives

Cerebral visual impairment (CVI) comprises a heterogenous group of brain-related vision problems. A core outcome set (COS) represents the most important condition-specific outcomes according to patients, carers, professionals and researchers. We aimed to produce a COS for studies evaluating interventions for children with CVI, to increase the relevance of research for families and professionals and thereby to improve outcomes for affected children.

Design

We used methods recommended by the COMET (Core Outcome Measures in Effectiveness Trials) Initiative. These included a proportionate literature review of outcomes used in previous studies; qualitative interviews with children and families; a two-round Delphi survey involving parents, children and professionals and a consensus meeting to ratify the most important outcomes.

Setting

Telephone interviews and online Delphi surveys of participants who all lived in UK or Eire

Participants

Eighteen parents and 6 young people were interviewed. Delphi participants (n=80 did both rounds) included professionals working with children who have CVI (teachers, orthoptists, ophthalmologists, optometrists, qualified teachers for visually impaired (QTVI), family members (parents and siblings) and affected children.

Results

The literature review included 13 studies yielding 37 outcomes. Qualitative interviews provided 22 outcomes. After combining and refining similar items, the first round contained 23 outcomes and the second 46. At the consensus meeting, five attendees recommended 28 outcomes for inclusion in the CVI COS, of which 15 were ratified as most important, including 4 related to vision; 1 to family wellbeing; 1 to adults around the child being informed about CVI and the rest to the child's abilities to engage with people and surroundings.

Conclusions

Good engagement from participants led to the development of a COS. Future research will be useful to identify the best ways to measure COS items and potentially to update this COS as more interventions for CVI are developed.

297 words

Keywords: CVI, Children, Core outcome set, Paediatric

For peer review only

ARTICLE SUMMARY

Strengths and Limitations of the study

Robust tried and tested methods advocated by the COMET Initiative were used to propose a core outcome set for evaluative trials and routine practice in hospitals and schools on childhood cerebral visual impairment (CVI)

Parents, children and professionals in the UK and Eire took part in the process as interviewees, participants in a Delphi survey and/or consensus meeting and suggested several of the outcomes included in the final COS

The inclusion of a self-selecting sample means the precise criteria used to diagnose the participating children with CVI were not sought which may limit the generalisability of the findings, as well as the location being restricted to the UK and Eire

The COS are based on ratings by children, families and professionals, therefore the core outcomes identified may not reflect the views of researchers or journal editors who were not involved.

Several outcomes were broad concepts and further research is needed to determine how to measure the COS.

For peer review only

INTRODUCTION

Cerebral Visual Impairment (CVI) refers to a range of brain-related impairments of vision [1-3]. Although there is not yet an agreed definition for CVI, a recent review has suggested that CVI be regarded as a verifiable impairment of vision that is not attributable to ocular or anterior visual pathway (optic nerve) disorders[4]. However different groups and centres take different views on which visual disorders can be described as CVI [3, 5] and in particular there is variation in the levels of visual acuity loss that different authors describe as consistent with CVI [4]. There are not at present and nationally or internationally agreed guidelines for the management of CVI in children and clinical practices vary according to location, both within and between countries.

A core outcome set (COS) represents the most important condition-specific outcomes that reflect the priorities of patients, carers, professionals and researchers. Further work is then required to establish "how" to measure the items in the COS. Use of a COS ensures research findings are relevant to health service users, ie patients and carers, as well as to healthcare professionals. A COS facilitates synthesis across studies and aggregation of data in meta-analyses or evidence syntheses, to develop a robust evidence base [6].

Guidelines exist to aid researchers in developing core outcome sets (CO_STAD[7])and reporting processes (CO-STAR[8]), which we have followed insofar as our resources allowed. The COMET (Core Outcome Measures in Effectiveness Trials) database describes established and ongoing COS and did not previously include a COS for CVI. The COMET recommended methods [9] include a literature review to discover which outcomes have already been measured in relevant studies, qualitative research to elicit the views of patients, families and carers; a Delphi survey (an online, iterative survey with anonymous participants [10]) in which stakeholders from patient and professionals groups rated outcomes by importance and finally a consensus meeting involving representatives of all stakeholder groups to discuss and ratify the final items in the COS. These methods have recently been used to produce COS the scope of which was the assessment of brain-related impairments of vision in adults who have suffered a stroke[11]. We aimed to produce a COS with a scope of studies evaluating interventions for children with CVI, so as to enhance the relevance for patients of future intervention studies and ultimately improve outcomes for children with CVI, rather than aiding professionals with diagnosis.

METHODS

Scope

This COS relates to children aged up to 18 years in the UK and Eire, and to interventions to help children with CVI in any setting, including evaluative trials and routine practice in hospitals and schools. We took an inclusive approach and included CVI as a broad concept, diagnosed by a relevant professional, to reflect (a) the current lack of consensus about which brain-related impairments should be included, and (b) variation in availability of detailed vision testing to elicit all the potential manifestations of CVI.

Ethical Approval

The study was registered with the COMET Initiative (http://www.comet-initiative.org/studies/details/1032). The study was approved by the University of Bristol Faculty of Health Sciences Research Ethics Committee (FREC ref: 58441). Written consent was documented for the interviews, from both parents and young people. Consent to the Delphi survey was implied by the completion and return of the questionnaires.

For peer review only

Patient and Public Involvement

We used a range of methods to involve patients and the public and these are summarised in Figure 1, using the GRIPP2 recommended headings [12]. We convened meetings with two group of families: one in the Bristol area whose children have CVI and the other with the PenCRU Family Faculty at University of Exeter Medical School. The PenCRU Family Faculty comprises parents whose children have various neurodevelopmental conditions and who regularly contribute to and advise on research studies. We sought their advice on practical details of interacting with children with neurodevelopmental conditions in the planned qualitative interviews. We presented our plans at the yearly meeting of a family support group for children with CVI (the CVI Society, www.cvisociety.org.uk) and families were invited to participate in the qualitative interviews or Delphi survey and/or invite other people to participate. Study details were posted on the CVI Society website and in their closed Facebook group.

We met with our Professionals Advisory Group which includes education and health professionals with an interest in CVI. They advised on the best ways to contact professionals e.g. by using dedicated email lists. Parents and 3 young people with CVI then advised on the wording of the outcomes included in the Delphi survey.

Stage 1: Literature Review

A proportionate review of the literature was carried out to identify outcomes that have been measured in evaluations of interventions to help children with diagnosed CVI or with brain-related vision impairments if the term CVI was not used. A search strategy was updated from a previous scoping review on interventions for CVI, [13] and the resulting set of papers was searched specifically for papers reporting controlled studies, before-and-after studies or service evaluations, involving children with CVI using any definition or brain-related vision problems. Searches were completed in Medline, Embase, PsychInfo and the Cochrane database of systematic reviews (search dates 1946 – May 2018). Papers that reported on eligible studies involving children up to 18 years were included, including papers reporting methods before results were obtained, since these contained outcomes relevant to the purpose of our review. Papers where it was not possible to separate adult from child data, where there was no description of the visual behaviour used to define the child as having CVI or brain-related vision problems and papers not in English (as no translation funds were available), were excluded.

A data extraction form was used to record all outcomes used in each included paper, whether primary or secondary, the instruments used, the study type and setting, participant characteristics, and data completion for each outcome. Also included was which domain the outcome mapped to, using the COMET taxonomy[14]. Data were independently extracted by two reviewers (CW and TG) and consensus was reached by discussion with a third reviewer.

For peer review only

Stage 2: Qualitative interviews with families where a child has CVI

Participants were children and young people (CYP) aged 6 - 18 years with a diagnosis (by a professional) of CVI, using any definition, and/or parents or carers of children with this diagnosis. To include a range of ages and physical capabilities, maximum variation purposive sampling was used to recruit families to a matrix including younger (6-11 years old) and older (12-18 years old) children, and those with and without a diagnosis of cerebral palsy. Families were recruited from three sources: from local Specialist teachers for Vision Impairment; from The Specialist Centre for Visual Impairment, Exeter (WESC Foundation), and from the CVI Society, a national parent support group for families of children with CVI (www.cvisociety.org.uk) as described above. Recruitment was also facilitated by snowballing information to other interested groups via from the initial contacts, including links to 'Moorvision' (https://www.moorvision.org/) via a local specialist school (the WESC Foundation specialist school for visual impairment).

Interviews were carried out in person either face-to-face or by telephone or video link. Parents were present for interviews with young people. All interviews were audio-recorded with consent. Topic guides covered what day-to-day family life living with CVI is like, including school, home, family and health and wellbeing; practicalities of living with CVI; support received and what would have helped in the past and what would help in the future. For interviews with young people, a range of creative activities were used to help elicit their views about what matters most to them, including drawing, colouring and tablet-based art activities.

The interviews were transcribed verbatim and systematic coding across the transcripts was used to identify outcomes for inclusion in the Delphi Survey. The full thematic analysis of these interviews exploring the impact of CVI on the daily lives of children and their families will be reported separately.

Stage 3: Delphi Process

A Delphi survey[10] was used for participants to rank the importance of the outcomes assembled from the systematic review and the interview data. There were two rounds to the survey and a subsequent face-to-face consensus meeting to ratify agreement on the most important outcomes.

Two groups of participants were invited to take part in the Delphi survey: professionals and families. Professionals included ophthalmologists, optometrists, orthoptists, paediatricians and specialist teachers (Qualified Teachers for Visual Impairment, QTVIs). Families included parents of children with CVI and children living with the condition. Participants were recruited via email lists for local and national organisations, local schools, specialist teachers and patient support groups who advertised the survey to professionals and families. We also invited those families who took part in the qualitative work to take part in the Delphi survey if they wished, but this was not expected or mandatory. Professionals who responded to flyers or email invitations were sent formal invitations to the survey with a link for completion online.

DelphiManager (a web-based Delphi survey data management system developed by COMET) was used to facilitate the construction, administration and analysis of the surveys. The first round of the survey presented the initial list of outcomes compiled using the literature review and interviews. Respondents were asked to rank the importance of measuring each outcome in CVI research using a 9-point scale, where 9 was "critical to include" and 1 was "not important at all". We also invited respondents in the first round to add any additional outcomes that they felt should be included.

In the second round, respondents were shown the median ranking and range of rankings by each group, for each outcome and were reminded of their own ranking. They were given the opportunity to rank again items from the first round, and the additional items that had been suggested.

Stage 4: Consensus workshop

For peer review only - http://bmjopen.bmj.com/site/about/guidelines.xhtml

A workshop was held with attendees from both groups in Bristol, to ratify agreement on the most highly-ranked outcomes. We presented the three lists of outcomes: consensus 'in' with more than 70% from both groups ranking them as 7-9, consensus 'out' with more than 70% from both groups ranking them as 1-3, and partial or no agreement with less than 70% from either group ranking them as 7-9. Attendees discussed the inclusion or exclusion of the partial or no agreement list first, then moved on to discussing the rankings for all included outcomes and agreed a full list for the COS, after combining related outcomes to reduce overlap. Finally, attendees were asked to indicate their own personal "top 10" outcomes for inclusion. A shorter COS list was agreed containing all outcomes that at least one person selected for their personal top 10. With permission, the consensus meeting discussions were recorded.

The Protocol for the development of this COS is included in Supplementary Materials.

RESULTS

For peer review only

We followed our Protocol, but timing was later due to delays starting. For this reason we conducted 2 not 3 rounds of the Delphi survey.

Stage 1: Literature Review

5155 titles and abstracts were extracted from four databases. These were searched for evaluative studies and 22 were selected for full text review. Of these, 13 papers (10 studies) met the inclusion criteria and data were extracted. Three papers[15-17] reported on the use of bifocal spectacles for children with reduced accommodation and used visual outcomes (near vision, accommodative amplitude, preferred working distance) and educational outcomes (reading tests). Three papers [18-20]reported outcomes after treatments for symptomatic convergence insufficiency and they used visual (nearpoint of convergence, ability to fuse images, symptom score), educational (reading tests) and behavioural outcomes (child behaviour checklists). Two papers investigated the effects of treatments for children with reduced visual attention in the context of a diagnosed attentional disorder (ADHD): one used visual fields as the outcome[21] and one a score for visual attention[22]. Two papers investigated the effects of interventions to improve visuomotor skills: one involved prisms in children with hemispatial neglect and used visually-guided pointing as the outcome [23]and one used eye movement training and used eye movement, arms movement and ball-catching accuracy as outcomes[24]. The remaining papers investigated the effects of intraventricular stem cell transplant on visual behaviour[25]; music on attention to task[26] and a parenting programme on parent behaviour [27], all involving children with severe visual impairment. Thirty-seven individual outcomes were identified from the 13 papers in the review.

Stage 2: Qualitative interviews with families where a child has CVI

Families were from England (n=16), Scotland (n=1) and Northern Ireland (n=2) and one family lived in Eire. Twenty four interviews were completed: eighteen with parents/carers and six with children with a parent also present. Eight parent/carer interviews took place by telephone, ten interviews were face-to-face.

Overall, children/young people's ages ranged from 3 to 17 years. Ten children were under the age of 11, 6 were between 11-16 years of age and one was aged 17 years. The six children who took part in separate interviews were aged 6-17 years (2 were in the younger group of 6–11-year-olds and 4 were in the older group of 12-18 year olds). Of the six interviews conducted with children or young people, one used video call, one was by telephone and 4 were face to face. Other diagnoses were recorded as described by parents and included premature birth, cerebral palsy; chromosomal abnormalities; epilepsy; global developmental delay and autism.

From the analysis, 22 outcomes were identified across four themes of: (1) assessment and understanding of implications of CVI for CYP and families, (2) education, (3) family life, and (4) psychological wellbeing and quality of life.

All outcomes from the literature review and interviews were coded to the domains of the COMET taxonomy and these were used as section headings in the survey. We also included outcomes previously identified in a COS for children with neurodisability [28]. These were communication, emotional wellbeing, pain, sleep, mobility, self-care, independence, mental health, community and social life, behaviour, toileting and safety. Overlapping items were combined to reduce participant burden as much as possible. A final set of 23 discrete outcome domain items was included in the first round of the Delphi survey (Table 1). After discussion to combine similar outcomes and remove duplicate items, the 23 outcomes included in the first round included 5 relating to visual functions that came from the proportionate review of the literature, 8 outcomes relating to general health and abilities from the neurodisability COS and 10 outcomes from the results of the qualitative interviews.

Stage 3: Delphi Process

In total there were 126 participants in round 1 and 80 (64%) in round 2. The majority came from England (n=100), with the next largest group from Scotland (n=14) and small numbers from Wales (n=2) , N Ireland (n=8) and Eire (n=2). They comprised QTVIs (29, then 21); Consultant Ophthalmologists (11 then 5); Consultant Paediatricians (4 then 2); Orthoptists (8 then 5); Habilitation Officers (8 then 5) Optometrists (3 then 2) and other professionals (17 then 12), plus the family members (46 then 28).

Twenty additional outcomes were suggested by participants in Round 1 of the survey, some from CYP, some from adult family members and some from professionals. These included 5 vision-related outcomes including sensitivity to light, ability to see colour and to pick out features in crowded scenes; other sensory outcomes related to hearing; more outcomes relating to social functioning and family wellbeing and one outcome related to adults around the child understanding CVI.

A total of 43 outcomes were therefore ranked in round 2. Table 1 shows the initial list of 23 outcomes included for ranking by participants as well as the 20 additional outcomes suggested by participants, that were ranked in round 2.

Table 1. Outcomes rated in Rounds 1 and 2 of the Delphi Survey of outcomes to include in a Core Outcome Set for Childhood CVI

Rounds	Outcome	Description given in survey	COMET taxonomy Domain
1&2	**Ability to see things at a distance of at least 3 metres**	**For example looking at an eye chart with letters or pictures, with glasses if appropriate, visual acuity**	**Eye outcomes**
1&2	**Ability to focus on near things**	**For example toys, people's faces, letters or pictures, with glasses if appropriate**	**Eye outcomes**
1&2	**Ability to keep both eyes together on a near target**	**For example when reading, doing artwork, or using a computer or tablet**	**Eye outcomes**
1&2	**Ability to recognise visual targets**	**For example in picture books or on worksheets, adapted as necessary**	**Eye outcomes**
1&2	**Ability to track moving objects**	**For example follow the movements of other children, or animals or cars or balls**	**Eye outcomes**
1&2	**Mental health**	**For example, anxiety, depression, behavioural problems, self harm**	**Mental health including psychiatric problems**

For peer review only - http://bmjopen.bmj.com/site/about/guidelines.xhtml

1&2	Child's Emotional wellbeing	For example child's level of confidence, happiness etc	Emotional functioning/wellbeing
1&2	Child's Self-perception	For example how child thinks of themselves, self-esteem	Emotional functioning/wellbeing
1&2	Sleep	Sleeping during the night or at appropriate times	General physiological/clinical
1&2	Tiredness/Fatigue	For example during the day, not just at night; lack of stamina, whatever the cause	General physiological/clinical
1&2	Mobility	For example child able to move around their physical environment, including physical obstacles, within the limits of any motor impairment or disability	Physical functioning
1&2	Self-care	For example washing, feeding and dressing, both practical and social aspects	Physical functioning
1&2	Independence	Able to go out and about and/or carry out activities of daily living appropriate to age and development, in and out of the home	Physical functioning
1&2	Safety	Awareness of danger (physical and social), appropriate to age or development, in and out of the home	Physical functioning
1&2	Toileting	As appropriate to physical abilities, including physical and social aspects	Physical functioning
1&2	Route-finding	Finding way around in the community and in both familiar and new settings e.g. school	Physical functioning
1&2	Communication	For example with family, friends and others; verbal and non-verbal	Social functioning
1&2	Community and social life	For example taking part in clubs, sport, parties, other leisure activities or entertainments	Social functioning
1&2	Behaviour	For example managing emotions at home, school and in the wider community, self regulation	Social functioning
1&2	Play	Ability to play, be part of playful activities, on own or with others	Social functioning
1&2	Learning	Whether at school or home-taught, learning and applying knowledge, attention and concentration	Cognitive functioning
1&2	Financial costs	For example costs incurred by parents; working time lost	Societal/carer burden
1&2	Family wellbeing	For example parental emotional well-being, family functioning	Societal/carer burden
2 only	Consistency of visual performance	For example, changes in ability to process visual information when tired, throughout the day	Eye outcome
2 only	Functional 3D skills	For example, making models in Lego, or craft modelling, doing jigsaw puzzles, dressing and feeding themselves, if appropriate	Eye outcomes
2 only	Ability to see colour		Eye outcomes
2 only	Sensitivity to light	For example, may need additional lighting or reduced lighting, or tinted glasses	Eye outcomes
2 only	Ability to deal with crowded or cluttered scene	For example, finding clothes, toys and people in cluttered or crowded spaces	Eye outcomes
2 only	Ability to watch TV, films or run computer games		Eye outcomes
2 only	Hearing	Being aware of sounds	General physiological/clinical
2 only	Auditory Understanding	For example, knowing the direction of sound	General physiological/clinical
2 only	Appreciation/enjoyment of music	Child enjoys listening to/experiencing music in whatever form	Social functioning

For peer review only - http://bmjopen.bmj.com/site/about/guidelines.xhtml

2 only	**Pain**	**For example discomfort due to positioning, headaches or other sources of pain or discomfort**	**General physiological/clinical**
2 only	**Child's understanding of own needs and their condition**	**For example, knowing how CVI and other comorbidities affect them and what helps or hinders their daily lives, including being aware of visual field loss**	**Emotional functioning/wellbeing**
2 only	**Child's resilience**	**For example, child uses appropriate coping mechanisms or strategies to manage CVI and other conditions**	**Emotional functioning/wellbeing**
2 only	**Reading or accessing books**		**Cognitive functioning**
2 only	**Ability to cope with moving environment**	**For example, car travel, train travel, bus travel**	**Physical functioning**
2 only	**Environmental adaptations**	**Appropriate adaptations available including: learning materials, support with travel and transport**	**Physical functioning**
2 only	**Environmental adaptations are acceptable to the child**	**Child can or will access/use environmental adaptations as provided, for example, learning materials, support with travel and transport**	**Physical functioning**
2 only	**Managing in crowded environments**	**For example, in shops, cinemas, streets**	**Social functioning**
2 only	**Social life/relationships**	**For example, ability to make and sustain friendships and relationships**	**Social functioning**
2 only	**General Health**	**Self or proxy rated health, for example, 'how are you in yourself?'**	**General physiological/clinical**
2 only	**Relevant adults aware of CVI**	**Teachers, family etc know what CVI is and what helps the child**	**Social functioning**

Following the second round, the proportion of people ranking outcomes as of high importance with a ranking of 7-9 out of 9 was calculated. Of the total of 43 outcomes, 25 were "in" initially as >70% of each stakeholder group had ranked them as 7-9. Table 2 summarises the ranking scores and shows outcomes in red where both groups ranked them lower than 70%, in green where both ranked higher than 70% and in orange where one group ranked higher and one lower.

Table 2. Results of Delphi survey: percentages of 85 respondents who ranked the outcome with a score of 7-9 indicating they were important to include in the Core Outcome Set for Childhood CVI

Outcome	Domain	Families N = 30	Professionals N = 55
**Outcomes in first round**			
**Ability to see things at a distance of at least 3 metres**	Eye Outcomes	68%	67%
**Ability to focus on near things**	Eye Outcomes	87%	87%
**Ability to keep both eyes together on a near target**	Eye Outcomes	62%	53%
**Ability to recognise visual targets**	Eye Outcomes	97%	95%
**Ability to track moving objects**	Eye Outcomes	97%	76%
**Mental health**	Mental health	77%	83%
**Child's Emotional wellbeing**	Emotional functioning/wellbeing	83%	100%
**Child's Self-perception**	Emotional functioning/wellbeing	77%	87%
**Sleep**	General physiological/clinical	73%	76%
**Tiredness/Fatigue**	General physiological/clinical	87%	85%
**Mobility**	Physical functioning	93%	81%
**Self-care**	Physical functioning	80%	69%
**Independence**	Physical functioning	83%	89%

For peer review only - http://bmjopen.bmj.com/site/about/guidelines.xhtml

**Safety**	Physical functioning	97%	91%
**Toileting**	Physical functioning	60%	54%
**Route-finding**	Physical functioning	77%	65%
**Communication**	Social functioning	94%	96%
**Community and social life**	Social functioning	83%	87%
**Behaviour**	Social functioning	77%	72%
**Play**	Social functioning	80%	87%
**Learning**	Cognitive functioning	94%	88%
**Financial costs**	Societal/carer burden	53%	41%
**Family wellbeing**	Societal/carer burden	76%	90%
**Outcomes suggested by participants in Round 1**			
**Consistency of visual performance**	Eye Outcomes	80%	63%
**Functional 3D skills**	Eye Outcomes	78%	53%
**Ability to see colour**	Eye Outcomes	33%	26%
**Sensitivity to light**	Eye Outcomes	73%	63%
**Ability to deal with crowded or cluttered scene**	Eye Outcomes	83%	77%
**Ability to watch TV, films or run computer games**	Eye Outcomes	57%	39%
**Hearing**	General physiological/clinical	80%	68%
**Auditory Understanding**	General physiological/clinical	94%	68%
**Appreciation/enjoyment of music**	General physiological/clinical	50%	41%
**Pain**	General physiological/clinical	76%	72%
**Child's understanding of own needs and their condition**	Emotional functioning/wellbeing	64%	68%
**Child's resilience**	Emotional functioning/wellbeing	83%	81%
**Reading or accessing books**	Eye Outcomes	63%	54%
**Ability to cope with moving environment**	Eye Outcomes	94%	72%
**Environmental adaptations**	Physical functioning	87%	73%
**Environmental adaptations are acceptable to the child**	Physical functioning	89%	87%
**Managing in crowded environments**	Social functioning	80%	63%
**Social life/relationships**	Social functioning	87%	90%
**General Health**	General physiological/clinical	73%	67%
**Relevant adults aware of CVI**	Delivery of care	94%	90%

Stage 4: Consensus workshop

Five people, two family representatives and three professionals, attended the consensus meeting in Bristol in March 2019. After a discussion, including of the 9 outcomes where there was uncertainty, some similar items were combined and 28 outcomes were listed for inclusion in a final COS. Participants were then asked to vote on their ten most important outcomes. From this, a shorter COS list was produced, containing all the outcomes that at least one person put in their own top ten. The full list of all agreed outcomes is shown in Table 3. The top 15 of these comprise a short COS, of outcomes at least one person put in their personal "top ten".

Table 3. Results from Consensus meeting exercise: full list of items for inclusion in the Core Outcome Set for Childhood CVI and number of votes for participants' personal "top ten", at Consensus meeting

Number	Outcome	N who included outcome in their top ten (max 5)
1	Community/Social life and Relationships*	5

For peer review only - http://bmjopen.bmj.com/site/about/guidelines.xhtml

2	Learning and accessing books or screens*	5
3	Child emotional wellbeing	4
4	Tiredness/fatigue	4
5	Consistency of visual performance*	4
6	Managing in crowded environments*	4
7	Relevant adults aware of CVI*	4
8	Ability to deal with crowded or cluttered scene*	4
9	Mobility	3
10	Communication	3
11	Child's understanding of their own needs*	3
12	Safety	2
13	Environmental adaptations*	2
14	Ability to recognise visual targets	1
15	Family wellbeing	1
16	Ability to focus on near things	0
17	Ability to keep both eyes together on a near target	0
18	Ability to track moving objects	0
19	Child's Self-perception	0
20	Sleep	0
21	Self-care	0
22	Behaviour	0
23	Play	0
24	Functional 3D skills*	0
25	Sensitivity to light*	0
26	Ability to watch TV, films or run computer games*	0
27	Pain*	0
28	Ability to cope with moving environment*	0

Figure 2 summarises the different stages of the process to identify the COS and the results obtained.

There were 4 vision-related outcomes in the shorter COS (consistency of visual performance, ability to pick out features in a cluttered scene, ability to cope in a crowded environment and ability to recognise visual targets) as well as the outcome that adults around the child understood about CVI. In the short COS, 8 outcomes were suggested in part or completely, by the participants in round 1 (proposers included CYP, adult family members and professionals). Four were outcomes from the neurodisability COS. Two outcomes were voted as in their top ten by all 5 attendees: "community/interpersonal relations" and "ability to learn including accessing books or screens".

DISCUSSION

This study brought together existing literature, families, professionals and us as researchers to reach a consensus on the most important outcomes to measure when conducting research into interventions to help children with CVI. There was good engagement by the participants at all stages and, although the consensus meeting numbers were small, a clear plan for the day and a committed group of individuals were able to find common ground within their respective varied perspectives. Participants identified some outcomes with borderline results in Round 2 as important to include and did not reject any outcomes that had been included on the basis of Round 2 results. Although the participants were necessarily self-selecting and therefore not representative of the whole group or population with CVI, the consensus meeting validated, extended and ratified the Delphi survey results.

The COS of 28 items includes 12 vision-specific outcomes relating to a child's visual abilities, supporting the value of a dedicated COS for children with brain-related vision impairments, as opposed to using a more general COS for children with neurodisability. Outcomes included being able to see targets in cluttered scenes, or when moving and consistency of visual performance. These aspects of visual performance are asked about in several questionnaires relating to CVI [29, 30] and the results of this exercise support the importance of asking about them when assessing a child with potential CVI. However, the majority of outcomes related to other domains in a child's life and their ability to engage with other people and their surroundings. Several of the outcomes in the full COS were originally identified in the COS for children with neurodisability, reflecting the range of co-morbidities that are

For peer review only - http://bmjopen.bmj.com/site/about/guidelines.xhtml

associated with CVI and were reported by our participants. The short COS featured 4 related to vision in real-world complex situations and the other 11 related to wellbeing, learning, interacting with the world and aspects of general health. This reflects the comments of the families in the interviews about the wide-ranging effects of CVI and indicates that professionals were also aware of the importance of non-visual outcomes.

This project was proportionate to the resources available and represents a first proposal of a COS for children with CVI, which is a relatively new field for clinical intervention studies. Our scope was the UK and Eire, and the children are likely have had a variety of visual problems as we did not have access to their medical records to check why or how they were diagnosed with CVI. However, presently, there is no agreed definition of CVI, beyond a consensus that the visual deficits involved cannot be explained by ocular or optic nerve pathology. Future research may explore more targeted COS for children with CVI who display particular characteristics such reduced visual acuity, or visuo-cognitive impairments or who fit defined CVI subtypes such as those recently described using a data-driven approach [31].

Many of the outcomes are themselves broad, multifaceted concepts such as “Community, social life and relationships” and further work is needed to identify available validated measures for each outcome if available. However, the consistency with which these concepts were ranked as important indicates their importance to try and address, in some way. Increasing awareness of CVI was an important outcome for families and this may change as the condition becomes better understood and as agreed guidelines for diagnosis and management emerge.

Limitations of the study include that the literature review was targeted to specific study designs and some relevant evaluative studies may have been missed. We included papers reporting interventions targeting brain-related vision problems in children and may have included studies that some readers would not consider as relevant to CVI. We did not examine qualitative studies or health-related quality of life studies in children with CVI that may have elicited additional outcome domains. We did include an established COS for children with neurodisability, plus multiple items derived from the interviews, and we gathered additional suggestions from participants in the first round of survey. We are likely therefore to have included most or all outcomes of key importance and excluded those that were not felt to be important, in the views of our participants.

We might have identified more outcomes if we conducted more interviews, however continuing to interview until no new themes emerge (data saturation) is not always appropriate[32]and we succeeded in our aim to elicit a rich set of outcomes with which to populate the first Delphi survey. There were few children who were interviewed and/or who took part in the Delphi survey and these were necessarily those who could self-report to a reasonable level. We may have under-represented the views of younger or less able children - however all had been diagnosed with CVI and could speak to their experience about their lives. Only a small number of our participants were available to attend the consensus meeting which may have limited the range of opinions. Nevertheless all five participants were fully engaged and contributed to all aspects of the discussion and decision-making. During the process there were repeated exercises to reduce overlapping or similar types of outcome. This was a subjective process and may have resulted in loss of some more specific outcomes that in other settings would be rated important. There is still a degree of overlap between the outcomes and another group may have refined them differently. However, there was strong agreement that both visual function-related outcomes and child-centred outcomes relating to life chances and quality of life, were of crucial importance to include. We did not include users of core outcome sets such as researchers and journal editors, so did not capture their preferences in the consensus. We welcome discussion with colleagues to build on our proposed COS, which is offered as a useful starting point that may be refined in future, when national and/or international consensus is reached on the diagnosis of CVI in children.

For peer review only - http://bmjopen.bmj.com/site/about/guidelines.xhtml

A strength of this work is the high level of engagement of participants, the inclusion of children as well as parents and the broad levels of agreement between families and professionals. We used a variety of ways to identify relevant candidate outcomes and followed the COMET process that has been optimised iteratively over time. Several of the outcomes in the full COS and the short COS were suggested by the participants during the process. This indicates the value of inviting suggestions from participants to gain perspectives not suggested by the literature review or the interviews, perhaps because the context of rating outcomes stimulated different ideas from the more general discussion topics in the interviews.

Conclusions

A first COS for intervention studies on childhood CVI has been proposed. More work is needed to identify which tools might best be used to measure these and some may have to be addressed qualitatively if no validated tools exist. Future COS research could also examine modifications of the full COS for more specified groups or interventions, i.e. with a more restricted scope, or any changes that might result from including COS users e.g. journal editors or whether a having larger number of attendees at a consensus meeting leads to different selections of outcomes. Future research into interventions to help children with CVI should include at least the short COS items if possible, to increase relevance for the research for families and professionals and to aid cross-study comparisons.

Acknowledgements

We are very grateful to the families and professionals who took part in this study, including the interviewees, and participants in the Delphi survey and our consensus meeting: Kirsty Goodman, Janet Harwood, Kate Murton, Grainne Ni Dhuill, and Penny Warnes. We are also grateful to the members of the PenCRU Family Faculty who advised.

Author Contributions

The study was designed and led by CW, with help from all authors. CM advised on all aspects, CB did the literature search, TG led on the participant interviews and RW, TG and AP led on the Delphi study. AP drafted the first version of the manuscript and all authors contributed to revisions and approved the final manuscript.

Funding

This research was funded by the National Institutes of Health Research (NIHR) as part of a Senior Research Fellowship for CW: SRF_2015_08_005.

Disclaimer

The views expressed in this publication are those of the author(s) and not necessarily those of the NIHR, NHS or the UK Department of Health and Social Care.

Competing Interests

The authors have no competing interests.

For peer review only

Data Sharing

Anonymised data will be shared on reasonable request.

For peer review only

Legend for Table 2:
Green= 70% or more of each group rated outcome as 7-9, so put forward for inclusion
Orange = 70% or more of one group only rated outcome as 7-9, so put forward for discussion at Consensus meeting
Red= fewer than 70% of respondents in each group rated the outcome as 7-9, so outcome not put forward for inclusion

Legend for Table 3:
Shaded rows are outcomes suggested in full or in part by respondents in the Delphi survey

Legend for Figure 1:
Box describing how Parents and children were involved in the study

Legend for Figure 2:
Flow chart indicating the process involved in deriving the COS

For peer review only

REFERENCES

1. Dutton, G.N. and L.K. Jacobson, *Cerebral visual impairment in children.* Semin Neonatol, 2001. **6**(6): p. 477-85.
2. Ortibus, E., E. Fazzi, and N. Dale, *Cerebral Visual Impairment and Clinical Assessment: The European Perspective.* Semin Pediatr Neurol, 2019. **31**: p. 15-24.
3. Hoyt, C.S., *Visual function in the brain-damaged child.* Eye (Lond), 2003. **17**(3): p. 369-84.
4. Sakki, H.E.A., et al., *Is there consensus in defining childhood cerebral visual impairment? A systematic review of terminology and definitions.* Br J Ophthalmol, 2018. **102**(4): p. 424-432.
5. McConnell, E.L., K.J. Saunders, and J.A. Little, *What assessments are currently used to investigate and diagnose cerebral visual impairment (CVI) in children? A systematic review.* Ophthalmic Physiol Opt, 2021. **41**(2): p. 224-244.
6. Williamson, P.R., et al., *Developing core outcome sets for clinical trials: issues to consider.* Trials, 2012. **13**: p. 132.
7. Kirkham, J.J., et al., *Core Outcome Set-STAndards for Development: The COS-STAD recommendations.* PLoS Med, 2017. **14**(11): p. e1002447.
8. Kirkham, J.J., et al., *Core Outcome Set-STAndards for Reporting: The COS-STAR Statement.* PLoS Med, 2016. **13**(10): p. e1002148.
9. Williamson, P.R., et al., *The COMET Handbook: version 1.0.* Trials, 2017. **18**(Suppl 3): p. 280.
10. Hasson, F., S. Keeney, and H. McKenna, *Research guidelines for the Delphi survey technique.* J Adv Nurs, 2000. **32**(4): p. 1008-15.
11. Rowe, F.J., L.R. Hepworth, and J.J. Kirkham, *Development of core outcome sets for vision screening and assessment in stroke: a Delphi and consensus study.* BMJ Open, 2019. **9**(9): p. e029578.
12. Staniszewska, S., et al., *GRIPP2 reporting checklists: tools to improve reporting of patient and public involvement in research.* BMJ, 2017. **358**: p. j3453.
13. Williams, C., et al., *How to help children with neurodevelopmental and visual problems: a scoping review.* Br J Ophthalmol, 2014. **98**(1): p. 6-12.
14. Dodd, S., et al., *A taxonomy has been developed for outcomes in medical research to help improve knowledge discovery.* Journal of Clinical Epidemiology, 2018. **96**: p. 84-92.
15. Nandakumar, K., et al., *Bifocals in Down syndrome study (BiDS): analysis of video recorded sessions of literacy and visual perceptual skills.* Clin Exp Optom, 2011. **94**(6): p. 575-85.
16. Nandakumar, K. and S.J. Leat, *Bifocals in children with Down syndrome (BiDS) - visual acuity, accommodation and early literacy skills.* Acta Ophthalmol, 2010. **88**(6): p. e196-204.
17. Stewart, R., J. Woodhouse, and L.D. Trojanowska, *In focus: the use of bifocal spectacles with children with Down's syndrome.* Ophthalmic and Physiological Optics, 2005. **25**: p. 514-522.
18. Borsting, G., et al., *Behavioral and Emotional Problems Associated With Convergence Insufficiency in Children: An Open Trial.* Journal of Attention Disorders, 2016. **20**: p. 836-844.
19. Convergence Insufficiency Treatment Trial Study, G., *Randomized clinical trial of treatments for symptomatic convergence insufficiency in children.* Arch Ophthalmol, 2008. **126**(10): p. 1336-49.
20. Scheiman, M., et al., *Effect of treatment of symptomatic convergence insufficiency on reading in children: a pilot study.* Clin Exp Optom, 2018. **101**(4): p. 585-593.
21. Martin, L., et al., *Visual fields in children with attention-deficit / hyperactivity disorder before and after treatment with stimulants.* Acta Ophthalmol, 2008. **86**(3): p. 259-64.
22. Vaisman, N., et al., *Correlation between changes in blood fatty acid composition and visual sustained attention performance in children with inattention: effect of dietary n-3 fatty acids containing phospholipids.* Am J Clin Nutr, 2008. **87**(5): p. 1170-80.
23. Riquelme, I., et al., *Use of prism adaptation in children with unilateral brain lesion: Is it feasible?* Res Dev Disabil, 2015. **43-44**: p. 61-71.

For peer review only - http://bmjopen.bmj.com/site/about/guidelines.xhtml

24. Miles, C.A., et al., *Quiet eye training aids the long-term learning of throwing and catching in children: Preliminary evidence for a predictive control strategy.* Eur J Sport Sci, 2017. **17**(1): p. 100-108.
25. Luan, Z., et al., *Neural stem/progenitor cell transplantation for cortical visual impairment in neonatal brain injured patients.* Cell Transplant, 2013. **22 Suppl 1**: p. S101-12.
26. Robb, S.L., *Music interventions and group participation skills of preschoolers with visual impairments: raising questions about music, arousal, and attention.* J Music Ther, 2003. **40**(4): p. 266-82.
27. Overbeek, M.M., et al., *The effectiveness of VIPP-V parenting training for parents of young children with a visual or visual-and-intellectual disability: study protocol of a multicenter randomized controlled trial.* Trials, 2015. **16**: p. 401.
28. Morris, C., et al., *Meaningful health outcomes for paediatric neurodisability: Stakeholder prioritisation and appropriateness of patient reported outcome measures.* Health Qual Life Outcomes, 2015. **13**: p. 87.
29. University, U. *CVI Resources*. Available from: https://www.ulster.ac.uk/research/topic/biomedical-sciences/research/optometry-and-vision-science-research-group/vision-resources/professionals/cerebral-visual-impairment-assessment.
30. Ortibus, E., et al., *Screening for cerebral visual impairment: value of a CVI questionnaire.* Neuropediatrics, 2011. **42**(4): p. 138-47.
31. Sakki, H., et al., *Visual function subtyping in children with early-onset cerebral visual impairment.* Dev Med Child Neurol, 2021. **63**(3): p. 303-312.
32. Braun, V. and V. Clarke, *To saturate or not to saturate? Questioning data saturation as a useful concept for thematic analytsis and sample size rationales.* Qualitatve Research into Sport, Exercise and Health, 2021. **13**(2): p. 201 - 216.

For peer review only

Figure 1. Box describing Patient and public involvement in Core Outcome Set for childhood CVI, using GRIPP2 headings

How was the development of the research question and outcome measures informed by patients' priorities, experience, and preferences?
Parents and patients told us about the wide-ranging spectrum of effects that they felt CVI had on their children's lives. The emphasised the importance of poor mental health for children with CVI but no support.

How did you involve patients in the design of this study?
Parents approved the approach recommended by the Comet Initiative and recommended including home-schooled children if possible.

Were patients involved in the recruitment to and conduct of the study?
Parents recruited interviewees and Delphi participants via word-of-mouth using a closed Facebook group and parent networks. Parents recommended ways to engage with child interviewees. Parents and children reviewed and revised the wording of the Delphi surveys.

How will the results be disseminated to study participants?
We will provide a summary of the process and the findings from the qualitative interviews, Delphi process and consensus meeting to all participants. We will present the findings to the yearly meeting of a parent support group (www.cvisociety), post a summary on our study website, present the findings at conferences and submit a paper to a peer reviewed journal.

For peer review only

Figure 2

Flowchart of development of Core Outcome set for Childhood CVI

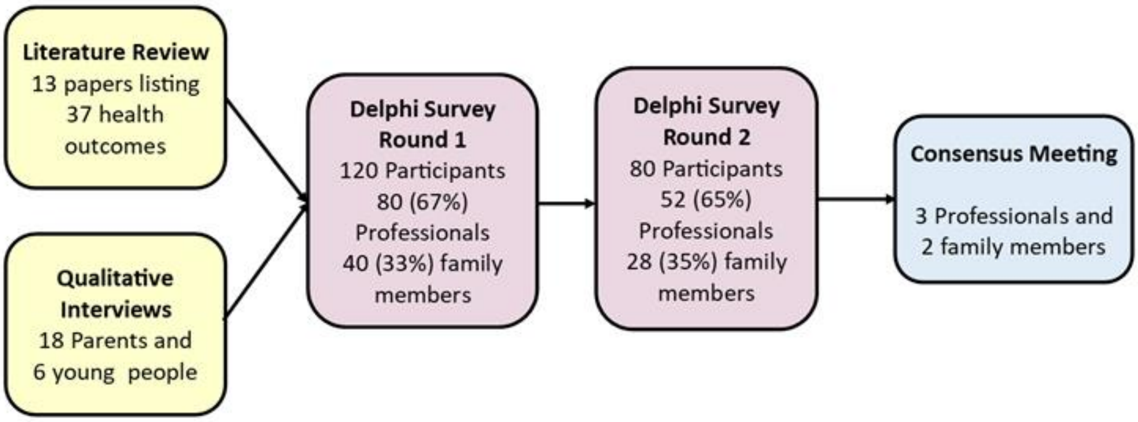

review only

University of BRISTOL

Research Protocol

The CVI Project: A Core Outcome Set for children with Cerebral Visual impairments (COS-CVI)

THE CVI PROJECT

NHS
National Institute for Health Research

Cathy Williams
& Anna Pease

Contents

Project Team 3

A Core Outcome Set for Children with Cerebral Vision Impairments (COS-CVI) 4

Background 5

Methods 6

Scope 6

Public Involvement 6

Phase 1. Identifying Candidate Outcomes 6

Proportionate Systematic Review 6

Qualitative work 7

Phase 2. Rating the importance of Candidate Outcomes using a Delphi Survey 9

Panels 9

Software, nature and ordering of questions 9

Number of rounds and nature of feedback given 9

Consensus workshop 10

Discussion and implementation 11

Gantt Chart for Core Outcome Set Development 12

Figure 1: Core Outcome Set Development Flowchart 13

References 14

For peer review only

Project Team

Ms Cathy Williams
Post: Reader in Paediatric Ophthalmology
Email: Cathy.Williams@bristol.ac.uk
Dept: Centre for Child and Adolescent Health, School of Social and Community Medicine
Address: University of Bristol, Oakfield House, Oakfield Grove, Bristol, BS8 2BN
Tel: 0117 3313388

Dr Anna Pease
Post: Senior Research Associate
Email: A.pease@bristol.ac.uk
Dept: Centre for Child and Adolescent Health, School of Social and Community Medicine
Official Address: University of Bristol, Oakfield House, Oakfield Grove, Bristol, BS8 2BN
Tel: 0117 331 3310

Dr Trudy Goodenough
Post: Senior Research Associate
Email: Trudy.Goodenough@bristol.ac.uk
Dept: Centre for Child and Adolescent Health, School of Social and Community Medicine
Official Address: University of Bristol, Oakfield House, Oakfield Grove, Bristol, BS8 2BN
Tel: 0117 331 3310

Ms Rose Watanabe
Post: Executive Assistant
Email: rose.watanabe@bristol.ac.uk
Dept: Centre for Child and Adolescent Health, School of Social and Community Medicine
Official Address: University of Bristol, Oakfield House, Oakfield Grove, Bristol, BS8 2BN
Tel: 0117 331 4005

Dr Christopher Morris
Post: Senior Research Fellow
Email: Christopher.Morris@exeter.ac.uk
Dept: Child Health Group Peninsula Cerebra Research Unit for Childhood Disability Research (PenCRU)
Official Addess: University of Exeter Medical School, South Cloisters, St Luke's Campus, Exeter, EX1 2LU
Tel: 01392 262980

Ms Cath Borwick
Post: Research Engagement Librarian - Medical Faculties
Email: cath.borwick@bristol.ac.uk
Dept: Medical Library
Official Address: Medical Sciences Building, University of Bristol, Bristol BS8 1TD
Tel: (0117) 331 1504 or 394 1079

Funding Source: National Institute of Health Research (NIHR) Senior Research Fellowship Award

For peer review only - http://bmjopen.bmj.com/site/about/guidelines.xhtml

A Core Outcome Set for Children with Cerebral Vision Impairments (COS-CVI)

Abstract

In the current climate of financial challenges and limited resources, the importance of best use of available funds is paramount. Core outcome sets are condition-specific sets of outcomes that are developed by and represent the concerns of a range of stakeholders including patients, carers, professionals and policy makers. The aim of this project is to develop a COS for use in evaluative research on interventions that aim to improve outcomes for children with brain-related vision impairments, commonly known as cerebral visual impairments or CVI.

Methods

This project is nested within a 5-year programme of work investigating the prevalence, impact and effectiveness of support for primary school age children with CVI. We will use methods recommended by the COMET Initiative (Core Outcome Measures in Effectiveness Trials) including a proportionate review of what's been measured before; qualitative work to bring in the voices of children and families; a Delphi survey involving parents, children, professionals in medicine and teaching and therapists and a consensus meeting to determine the most important outcomes. We will then review the available tools and decide how best to measure the selected outcomes.

Discussion and Implementation

Our methods are proportionate to the resources available in the 5-year programme and we will reflect and discuss any consequent limitations of this on our findings. We will then use the COS and the tools that best measure the selected outcomes in a feasibility cluster-randomised controlled trial (fcRCT). Outcomes from the fcRCT will include the acceptability and practicality of the COS and these may be useful for further development of the COS.

For peer review only - http://bmjopen.bmj.com/site/about/guidelines.xhtml

Background

Brain-related vision impairments, commonly known as cerebral visual impairments or CVI, are frequently reported in studies of patients who have suffered a form of injury to their central nervous syndrome or who are born with an abnormality in the brain structure or function.

CVI is an umbrella term used by many to include abnormalities in visual acuity, or contrast sensitivity or colour; ocular motility; visual field and the conscious and unconscious filtering or processing of visual input (1). However, in the current World Health Organisation (WHO) classification of diseases ICD10, only codes for acuity and field loss are used in the description of "visual impairment (VI)" and consequently in many countries, including the UK, certification of VI involves only those visual functions (2). There is not as yet a consensus within the UK or internationally as to how other impairments of visual functioning, such as eye movements or vision processing, should be characterised. The new ICD classification ICD11 is likely to contain addition codes for complex vision impairment affecting functions such as reading or navigation and this may aid future research and resource allocation (3). In the meantime, there are very few studies to guide parents or vision professionals or therapists on how much of a person's difficulties might be attributable to CVI, nor on how best to help them.

Individuals most at risk of CVI are those with childhood-onset neurodevelopmental disorders (1), and adults who develop neurological disorders particularly including stroke and dementia (4). Some of the problems relating to having CVI are likely to be present at all ages. Children with CVI have additional specific needs and problems relating to their development from infancy to adulthood and their need for education whilst adults who acquire their neurological problems later in life may have different problems relating to employment and adjustment to their new impairment.

Currently there are 17 projects registered in the "Eyes and Vision" section of the COMET database (5). All but two of them focus on adults rather than children. One of the adult projects is however specifically on CVI after stroke and will be contacted as there may be overlap between child and adult outcomes after CVI.

As part of establishing to what extent children with CVI might be helped by interventions in a future RCT, we will therefore develop a specific COS for children with CVI. We will take an inclusive approach and include CVI however diagnosed, to reflect (a) the current lack of consensus about what brain related impairments should be regarded as visual and (b) variation in availability of detailed vision testing to elicit all the potential manifestations of CVI.

For peer review only - http://bmjopen.bmj.com/site/about/guidelines.xhtml

Methods

We will register the study with the COMET Initiative. We will seek ethical committee permission through the University Faculty Research Ethics Committee (FREC).

Scope

We will include children of school-age up to 18 as they will be able to contribute their perspectives relating to their own recent past as well as their present, although the future fcRCT will be focussing on primary school children. We will consider outcomes of interventions to help children with CVI in any setting, including evaluative trials and routine practice in hospitals and schools. Our primary aim however is to arrive at an agreed COS for use in the planned fcRCT. We will use the PICO format to specify the scope of the study. The scope is participants in the UK and Ireland, though broader generalizability internationally could be explored subsequently.

Public Involvement

We already have had input from a local group of families whose children have CVI and from the PenCRU "Family Faculty" coordinated by University of Exeter. These are a group of parents whose children have a range of neurodevelopmental conditions. We will ask their advice on practical details of interacting with children with neurodevelopmental conditions in the planned qualitative interviews. They will also be consulted on interpreting the results and disseminating the findings.

Phase 1. Identifying Candidate Outcomes

Proportionate Systematic Review

We will review the literature to see which outcomes have been measured in interventions to help children with CVI.

Search strategy
We will include directly assessed, patient-reported and proxy-reported outcomes for children with CVI according to any definition made by a relevant professional (eg a vision specialist, a neuropsychologist or a doctor.

Types of Studies
Any interventional study that aims to improve outcomes for children with CVI as defined above

Types of Participants
Children up to 18 with CVI as defined above

For peer review only - http://bmjopen.bmj.com/site/about/guidelines.xhtml

Inclusion criteria
We will include studies that report on children with what the study reports as CVI, however defined. We will include experimental studies and observation studies that describe associations with CVI defined by a relevant professional

Exclusion criteria
We will exclude studies where it is not possible to separate adult from child data, where there is no description of the visual behaviour used to define the child as having CVI and studies not in English.

Data extraction
We will record each type of outcome recorded in included papers and the instrument used to measure or ascertain the outcome. We will record study type and participant characteristics as well as numbers of respondents and non-respondents, or non-applicable responses, for each outcome. Data will be extracted by two reviewers and compared and consensus reached by discussion with a third reviewer.

Synthesis
We will list all outcomes from the papers in a database as well as the instruments used. We will code and organize them in two ways. Firstly, we will use the forthcoming taxonomy proposed by the COMET Initiative (6). Secondly, we will map them to the domains in the WHO International Classification of Functioning (ICF) insofar that is possible. These two models will be used to sort the outcomes as per Wilson and Cleary (7) into biological or functional measures; activity or participation measures, mental health states and quality of life.

We will submit draft coding and lists to our advisory panels for discussion and refinement.

Qualitative work

Aim
The aim of the qualitative work will be to "fill in the gaps" revealed by the systematic review and capture the language and perspectives of children and their families.

Participants
Participants will be children and young people aged 6 - 18 years with a diagnosis of CVI, using any definition, and/or parents or carers of children with this diagnosis.

For peer review only

Sampling method

To include a range of ages and physical capabilities, maximum variation purposive sample will be used to recruit families to a matrix including younger (6-11 years old) and older (12-18 years old) children, and those with and without a diagnosis of cerebral palsy.

Recruitment

We will recruit children and young people from three sources initially and will use a snowballing approach to recruit more if needed. Firstly, we will ask the local Specialist teachers for Vision Impairment to post a notice with information and contact details on their website. They will also notify individuals they think might be interested. We will visit a local school for children with visual impairment (The WESC Foundation, Specialist Centre for Visual Impairment, Exeter) and distribute notices and information. Thirdly we will give the same information to a national parent support group for families of children with CVI (www.cvisociety.org.uk).

Data collection – interviews

Interviews will be carried out in person, at school or at home as preferred or as convenient. Children will be interviewed with a trusted adult present (this can be a parent or member of school staff), after their parent or carer has been interviewed. All interviews will be audio-recorded with consent. A topic guide based on the systematic review and comments from the advisory groups and based on approaches already reported and successful with similar-aged children will be used (for example an activity discussing "a day in the life of a child with CVI"). Interviews will be expected to take 30-45 minutes for adults and 10-30 minutes for children. When interviewing children a range of creative activities will help us to elicit views of young people about what matters most to them. We will use drawings, stories and a tablet to support these approaches to follow the topic guide.

Interviews will continue with families until the point of diminishing returns, when little or no new perspectives are forthcoming and no new outcome domains are being identified. We expect to conduct interviews with approximately 30 families in total.

Analysis

A framework analysis consisting of five steps will be used. First familiarisation with the data will allow for submersion in the topic area. Secondly an initial framework will be applied by coding key issues in the transcripts. Third, the codes will be indexed into categories, fourth, thematic charts will be produced and compared by independent coders (members of the research team) and finally the charts will be interpreted into themes using key ideas and quotes from the original data.

For peer review only - http://bmjopen.bmj.com/site/about/guidelines.xhtml

Mapping to a framework

We will map the data obtained from the qualitative interviews into categories according to the two coding schemes (the COMET taxonomy and the ICF) described above.

Phase 2. Rating the importance of Candidate Outcomes using a Delphi Survey

We will carry out a Delphi survey to rate the importance of the outcomes assembled from the systematic review and the interview data. A Delphi survey is an iterative process of defining and refining importance of outcomes, using several rounds of surveys involving parents, children, professionals in medicine and teaching and therapists. The process ends with a consensus meeting to determine the most important outcomes.

Panels

We will invite individuals to participate in either of two panels:
1. Professionals (ophthalmologists, optometrists, orthoptists, paediatricians, therapists, teachers)
2. Parents and YP and children.

We will ask local and national organisations to advertise the survey to professionals. We will ask local schools, the Specialist teachers and patient support groups to advertise the survey to families including children themselves if they want to participate, potentially with the support of a parent or carer, or independently. We will also invite those families who have taken part in the qualitative work to take part in the Delphi if they wish, but this is not expected or mandatory. We will invite individuals willing to participate to register through an online system (COMET's Delphi Manager) or to contact the study team directly. We will record the professional role, years in role, institution and area of the country for professionals taking part. We will record age, ethnicity and diagnosis/es of affected children for the families taking part.

Software, nature and ordering of questions

We will use DelphiManager (a web based data management system developed by COMET) to facilitate the construction and delivery of our surveys. The software allows data to be extracted and consolidated at every round, as well as providing analysis of responses.

Number of rounds and nature of feedback given

For peer review only - http://bmjopen.bmj.com/site/about/guidelines.xhtml

Round 1: In the initial stage of the survey we will present the findings of the systematic review and we will ask respondents to rate the importance of each using a 9 point scale in which 1-3 relates to “less important” and 7-9 relates to very important. We will also ask respondents to add any additional outcomes that they feel should be included. Those items that score a median of 7 or above and/or 70% from either panel rating the outcome as 7-9 will be carried into round 2.

Round 2: Following the initial stage of rating the systematic review findings and generating further outcomes, we will add in the list of outcomes provided through the qualitative work (taking out any overlapping items from each stream) and present the complete list in Round 2. We will ask respondents to rate each item as before. For those items that have been rated previously, respondents will be shown the median scores and range for three groups; (1) the scores from professionals, (2) the scores from families and (3) the overall median score from both panels. Those items that score a median of 7 or above and/or 70% from either panel rating the outcome as 7-9 will be carried into round 3.

Round 3: In round 3, respondents will be shown their score from Round 2 as well as the median score and range from each panel who responded for each item. We will include items where there is agreement between panels that items are important and also items where there is polarisation (e.g. professionals scored high but families scored low) between panels and indicate this in the survey. Space will be provided for respondents to add their own comments on how they have rated items and to add any further considerations.

We will send each final survey for each round to the Faculty of Health Sciences Research Ethics Committee for information.

Consensus workshop

We will hold a consensus workshop with attendees from both panels in Bristol, to ratify agreement of the more important outcomes. We will highlight that the core outcomes are a minimum set for measuring change and that interventions may add in other outcomes as they see fit. We will present a list of outcomes identified in round 3 with a median score of 7 or above with 70% from either panel rating the outcome as 7-9. Research team members will facilitate the workshop. All UK participants will be invited to attend. Attendees will be shown the list of outcomes to be ratified prior to the workshop. The workshop will comprise three parts.

For peer review only - http://bmjopen.bmj.com/site/about/guidelines.xhtml

Part 1 will include introductions for participants and introduce the format for the workshop. This will include the aims of the day and the threshold for inclusion of items into the final core set.
Part 2 will see participants discuss and rate the importance of items in mixed stakeholder groups, with a facilitator for each group.
Part 3 will bring the participants back together to arrange the final set of outcomes into importance and will be asked to endorse the proposed final set.

Discussion and implementation

Once we have identified what to include in the fcRCT from the above process, we will review the most feasible way to collect data on these outcomes. This COS project is necessarily proportionate to the resources in the NIHR Fellowship award that funds it and additional work such as the development of new assessment tools, is outside the scope and resources available. We will use the COS obtained from the consensus meeting to inform selection of outcome measures in the fcRCT. We will collect data on the feasibility and acceptability of the data collection for these outcome measures and use this to guide any further developments of a COS for children with CVI.

Timescale

The Gantt chart on page 12 indicates the sequence of events and the originally planned timescale. The qualitative work will extend into July due to staff changes and a

For peer review only

Gantt Chart for Core Outcome Set Development

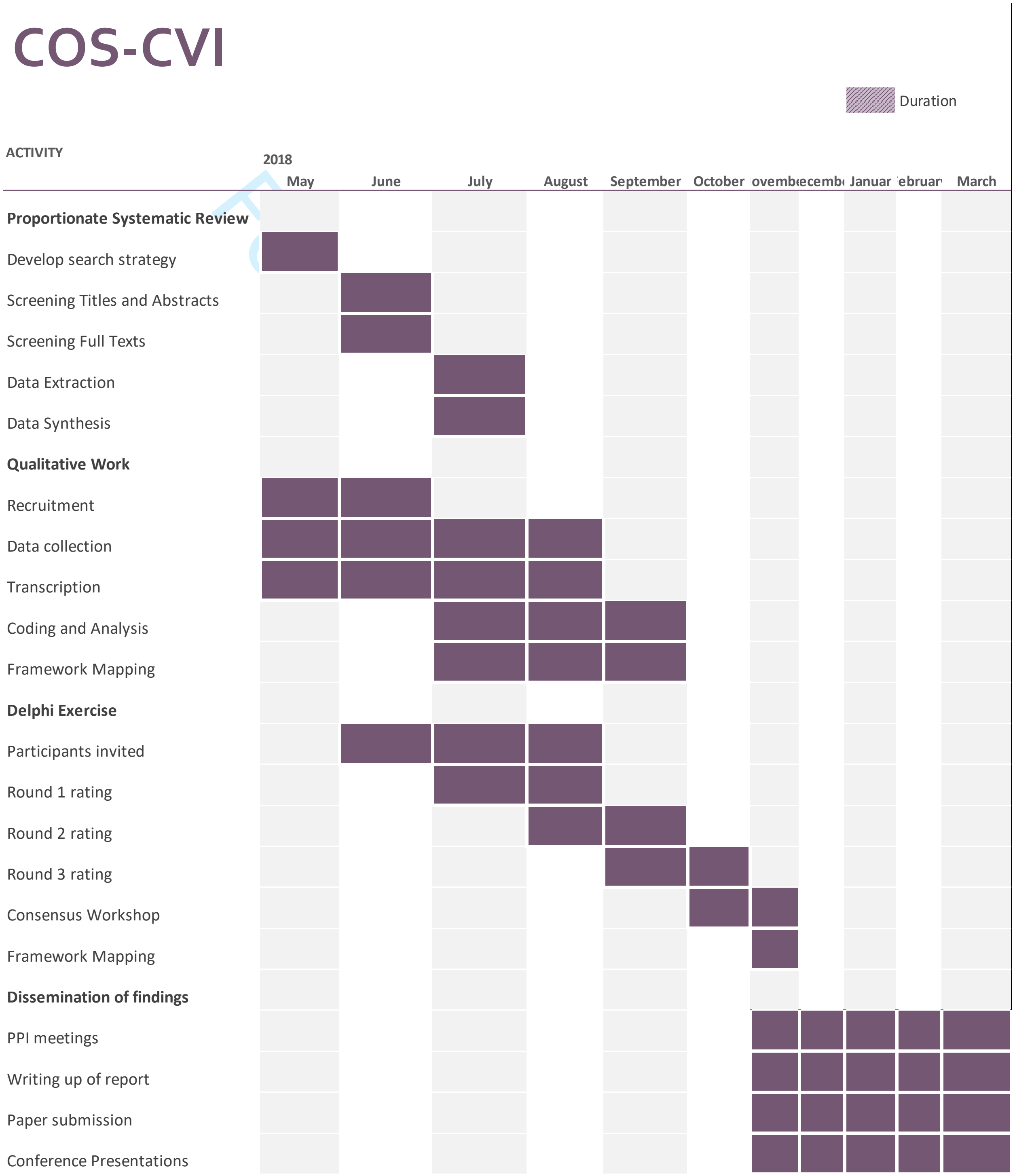

Figure 1: Core Outcome Set Development Flowchart

Phase 1: Proportionate Systematic Review

- Identify outcomes in RCTs involving children with CVI

Phase 2: Qualitative Interviews

- Capture perspectives and language of children with CVI and their families

Phase 3: Delphi Exercise

- Rate the importance of candidate outcomes
- Two panels - professionals and families

Round 1

1. Present outcomes from systematic review and rate on likert scale from 1 to 9
2. Provide feedback on ratings (optional)
3. Suggest new outcomes

Include Outcomes which score a median of 7 or above and/or 70% from either panel rating the

Round 2

1. Present outcomes from round 1 and qualitative interviews
2. Participants see own score, median for each panel and overall median score
3. Re-rate items from 1-9
4. Provide feedback on ratings (optional)

Include Outcomes which score a median of 7 or above and/or 70% from either panel rating the

Round 3

1. Present outcomes from round 2
2. Participants see own score, median for each panel and overall median score
3. Re-rate items from 1-9
4. Provide feedback on ratings (optional)

Consensus Workshop

1. Introductions and aims for the workshop
2. Break out groups to discuss and rate items
3. Consensus reached on core outcome set

Dissemination and Implementation

1. Report writing
2. Data collection methods for core outcomes reviewed
3. Select outcome measures for future fcRCT

Fig 1. Core Outcome Development Flowchart. Adapted from Tong et al. (2016)

For peer review only

References

1. Philip, S. S. and Dutton, G. N. (2014), Identifying and characterising cerebral visual impairment in children: a review. Clin Exp Optom, 97: 196–208. doi:10.1111/cxo.12155

2. https://www.gov.uk/government/publications/guidance-published-on-registering-a-vision-impairment-as-a-disability

3. https://icd.who.int/dev11/proposals/

4. Visual Perceptual Consequences of Stroke Fiona Rowe & VIS Group UK. Strabismus Vol. 17, 1,2009

5. http://www.comet-initiative.org

6. Trials. 2017 Jun 20;18(Suppl 3):280. doi: 10.1186/s13063-017-1978-4. The COMET Handbook: version 1.0.

7. Wilson IB, Cleary PD: Linking clinical variables with health-related quality of life: a conceptual model of patient outcomes. JAMA 1995, 273(1):59-65.

For peer review only

SECTION/TOPIC	ITEM No.	CHECKLIST ITEM	
**TITLE/ABSTRACT**			
Title	1a	Identify in the title that the paper reports the development of a COS	✓
Abstract	1b	Provide a structured summary	✓
**INTRODUCTION**			
Background and Objectives	2a	Describe the background and explain the rationale for developing the COS.	p4
	2b	Describe the specific objectives with reference to developing a COS.	p4
Scope	3a	Describe the health condition(s) and population(s) covered by the COS	p4
	3b	Describe the intervention(s) covered by the COS.	p4
	3c	Describe the setting(s) in which the COS is to be applied.	p4
**METHODS**			
Protocol/Registry Entry	4	Indicate where the COS development protocol can be accessed, if available, and/or the study registration details.	Supp
Participants	5	Describe the rationale for stakeholder groups involved in the COS development process, eligibility criteria for participants from each group, and a description of how the individuals involved were identified.	p5
Information Sources	6a	Describe the information sources used to identify an initial list of outcomes.	p5
	6b	Describe how outcomes were dropped/combined, with reasons (if applicable).	p6
Consensus Process	7	Describe how the consensus process was undertaken.	p6
Outcome Scoring	8	Describe how outcomes were scored and how scores were summarised.	p6
Consensus Definition	9a	Describe the consensus definition.	p6
	9b	Describe the procedure for determining how outcomes were included or excluded from consideration during the consensus process.	p6
Ethics and Consent	10	Provide a statement regarding the ethics and consent issues for the study.	p4
**RESULTS**			
Protocol Deviations	11	Describe any changes from the protocol (if applicable), with reasons, and describe what impact these changes have on the results.	p7
Participants	12	Present data on the number and relevant characteristics of the people involved at all stages of COS development.	p7-8
Outcomes	13a	List all outcomes considered at the start of the consensus process.	Table2
	13b	Describe any new outcomes introduced and any outcomes dropped, with reasons, during the consensus process.	Table 2
COS	14	List the outcomes in the final COS.	Table3
**DISCUSSION**			
Limitations	15	Discuss any limitations in the COS development process.	p13
Conclusions	16	Provide an interpretation of the final COS in the context of other evidence, and implications for future research.	p13
**OTHER INFORMATION**			
Funding	17	Describe sources of funding/role of funders.	p14
Conflicts of Interest	18	Describe any conflicts of interest within the study team and how these were managed.	p14, p11

doi:10.1371/journal.pmed.1002148.t001

1467x1100mm (72 x 72 DPI)
